# Alteration of L-Dopa decarboxylase expression in SARS-CoV-2 infection and its association with the interferon-inducible ACE2 isoform

**DOI:** 10.1371/journal.pone.0253458

**Published:** 2021-06-29

**Authors:** George Mpekoulis, Efseveia Frakolaki, Styliani Taka, Anastasios Ioannidis, Alice G. Vassiliou, Katerina I. Kalliampakou, Kostas Patas, Ioannis Karakasiliotis, Vassilis Aidinis, Stylianos Chatzipanagiotou, Emmanouil Angelakis, Dido Vassilacopoulou, Niki Vassilaki

**Affiliations:** 1 Laboratory of Molecular Virology, Hellenic Pasteur Institute, Athens, Greece; 2 Allergy and Clinical Immunology Unit, 2nd Pediatric Clinic, National and Kapodistrian University of Athens, Athens, Greece; 3 Department of Nursing, University of Peloponnese, Sparti, Greece; 4 First Department of Critical Care Medicine & Pulmonary Services, GP Livanos and M Simou Laboratories, National and Kapodistrian University of Athens Medical School, Evangelismos Hospital, Athens, Greece; 5 Department of Medical Biopathology, Medical School, University of Athens, Eginition Hospital, Athens, Greece; 6 Laboratory of Biology, Department of Medicine, Democritus University of Thrace, Alexandroupolis, Greece; 7 Institute for Bioinnovation, Biomedical Sciences Research Center “Alexander Fleming”, Athens, Greece; 8 Department of Diagnostics, Hellenic Pasteur Institute, Athens, Greece; 9 Aix Marseille Univ, IRD, IHU Méditerranée Infection, VITROME, Marseille, France; 10 Section of Biochemistry and Molecular Biology, Faculty of Biology, National and Kapodistrian University of Athens, Athens, Greece; Institut Pasteur, FRANCE

## Abstract

L-Dopa decarboxylase (*DDC*) is the most significantly co-expressed gene with *ACE2*, which encodes for the SARS-CoV-2 receptor angiotensin-converting enzyme 2 and the interferon-inducible truncated isoform *dACE2*. Our group previously showed the importance of DDC in viral infections. We hereby aimed to investigate *DDC* expression in COVID-19 patients and cultured SARS-CoV-2-infected cells, also in association with *ACE2* and *dACE2*. We concurrently evaluated the expression of the viral infection- and interferon-stimulated gene *ISG56* and the immune-modulatory, hypoxia-regulated gene *EPO*. Viral load and mRNA levels of *DDC*, *ACE2*, *dACE2*, *ISG56* and *EPO* were quantified by RT-qPCR in nasopharyngeal swab samples from COVID-19 patients, showing no or mild symptoms, and from non-infected individuals. Samples from influenza-infected patients were analyzed in comparison. SARS-CoV-2-mediated effects in host gene expression were validated in cultured virus-permissive epithelial cells. We found substantially higher gene expression of *DDC* in COVID-19 patients (7.6-fold; p = 1.2e-13) but not in influenza-infected ones, compared to non-infected subjects. *dACE2* was more elevated (2.9-fold; p = 1.02e-16) than *ACE2* (1.7-fold; p = 0.0005) in SARS-CoV-2-infected individuals. *ISG56* (2.5-fold; p = 3.01e-6) and *EPO* (2.6-fold; p = 2.1e-13) were also increased. Detected differences were not attributed to enrichment of specific cell populations in nasopharyngeal tissue. While SARS-CoV-2 virus load was positively associated with *ACE2* expression (r≥0.8, p<0.001), it negatively correlated with *DDC*, *dACE2* (r≤−0.7, p<0.001) and *EPO* (r≤−0.5, p<0.05). Moreover, a statistically significant correlation between *DDC* and *dACE2* expression was observed in nasopharyngeal swab and whole blood samples of both COVID-19 and non-infected individuals (r≥0.7). In VeroE6 cells, SARS-CoV-2 negatively affected *DDC*, *ACE2*, *dACE2* and *EPO* mRNA levels, and induced cell death, while *ISG56* was enhanced at early hours post-infection. Thus, the regulation of *DDC*, *dACE2* and *EPO* expression in the SARS-CoV-2-infected nasopharyngeal tissue is possibly related with an orchestrated antiviral response of the infected host as the virus suppresses these genes to favor its propagation.

## Introduction

Severe acute respiratory syndrome coronavirus 2 (SARS-CoV-2) is a positive-sense, single-stranded RNA virus, which leads to coronavirus disease 2019 (COVID-19). The mechanisms underlying virus-host interaction remain largely unknown. Symptoms range from fever, dry cough and dyspnea to the development of acute pneumonia, while neurological manifestations (loss of smell and taste, consciousness impairment, parkinsonism) have been also reported [1]. Severe aggravation in some patients has been associated with dysregulation of the immune response and induction of an inflammatory storm [2]. SARS-CoV-2 utilizes angiotensin-converting enzyme 2 (ACE2) as a receptor [3]. Apart from the full-length ACE2, an N-terminally truncated isoform, termed *delta*ACE2 (dACE2), has been recently reported, as a result of initiation from an alternative first exon, which is conserved only in primates [4, 5]. In contrast to ACE2, dACE2 protein does not bind the viral spike protein and *dACE2* is an interferon-stimulated gene (ISG), induced after SARS-CoV-2 infection. *dACE2* shows high expression in airway epithelial cells, in contrast to the lower levels of *ACE2*. The ratio of expression of the *dACE2* to *ACE2* is highest in nose and mouth and reduces in the lower respiratory tract. Although a transcriptome analysis of nasopharyngeal samples showed *ACE2* induction in COVID-19 patients [6], it has been hypothesized, but not experimentally addressed, that this is due to *dACE2* and not *ACE2* [4].

Analysis of a large number of human microarray datasets from various tissues revealed that the L-Dopa decarboxylase (*DDC*) gene exhibited the most statistically significant co-expression link with *ACE2* locus [7]. DDC catalyzes decarboxylation reactions, among them the conversion of L-3,4-dihydroxyphenylalanine (L-Dopa) to the immune-modulatory catecholamine dopamine [8, 9] and of 5-hydroxytryptophan to the inflammation and immunity regulator serotonin [10]. Both dopamine and serotonin act also as neurotransmitters providing a linkage between immune and neuron systems [11]. A physiological role for DDC in cell proliferation and apoptosis has been shown [12, 13]. In addition, our group was the first to unravel that *DDC* expression is associated with viral infections, and specifically has a negative relationship with hepatitis C (HCV) and Dengue (DENV) virus proliferation, in non-neuronal cells and in liver biopsies of HCV-infected patients [14]. Currently, there are no studies addressing the association of *DDC* expression with SARS-CoV-2 infection. However, viral proteins have been shown to interact with the catecholamine degradation enzymes monoamine oxidase A (MAOA) and catechol-O-methyltransferase (COMT) [15], and COMT has been reported as necessary for SARS-CoV-2 infection [16]. These findings, and the necessity of investigating the virus-host interactions for the emerging SARS-CoV-2, prompted us to extend our study on DDC in COVID-19 patients.

Both *DDC* and *ACE2* are regulated by hypoxia through mechanisms dependent on hypoxia-inducible factors (HIFs), as observed in various cell types, including airway epithelial cells [14, 17–22]. In nasal epithelial cells, HIF has been implicated in immunity and inflammation [23] and its target gene *EPO* has been regarded as a hypoxia marker [24]. However, prolonged hypoxia in cultured alveolar epithelial cells (A549) has been shown to induce apoptosis [25] and destabilizes HIF-1α mRNA, thus reducing the expression of its target genes [26]. SARS-CoV-2 infection results in airway and lung hypoxia that contributes to COVID-19 inflammation [27]. Cell culture data derived from alveolar and bronchial epithelial cells have revealed that although some of the HIF-α targets, such as VEGF, are upregulated by SARS-CoV-2, VHL that targets HIF-α to degradation is also upregulated [28]. In turn, HIF-1α activation inhibits SARS-CoV-2 infection in the these cells [22], at least in part due to lower *ACE2* expression and subsequent reduction of cell attachment of the viral spike protein [29]. In serum, lower levels of EPO have been detected in COVID-19 critical and deceased patient groups than in healthy ones [30].

In this study, we investigated the expression of *DDC*, *ACE2* and its interferon-inducible isoform *dACE2* in COVID-19 patients and in cultured SARS-CoV-2-infected cells. In parallel, the expression of other anti-viral host cell response related genes, specifically the viral infection- and interferon-inducible gene *ISG56* and the immune-modulatory, hypoxia-regulated gene *EPO* was also analyzed. A group of influenza-infected patients was studied in comparison, to examine if the detected alterations in gene expression are specific to COVID-19. Specifically, in nasopharyngeal swab samples from infected and non-infected subjects, we studied whether *DDC*, *ACE2*, *dACE2*, *ISG56* and *EPO* mRNA levels are associated with SARS-CoV-2 infection and viral RNA amounts. Moreover, the correlations among the mRNA levels of these genes were evaluated in nasopharyngeal swab and whole blood samples of COVID-19 patients. Finally, we determined in cultured mammalian epithelial cells (VeroE6, A549) the effects on gene expression exerted by SARS-CoV-2 virus and by hypoxia, a condition that has been associated with SARS-CoV-2 infection in airway epithelium.

## Materials and methods

### Patient samples

We tested a series of nasopharyngeal swab samples collected from patients with COVID-19 and from non-infected individuals as control. The inclusion criteria for patients were male or female adults having positive SARS-CoV-2 RNA PCR and showing no or mild COVID-19 clinical symptoms, including cough, sore throat, mild fever below 38 °C and loss of smell. The control group included individuals with a negative SARS-CoV-2 RNA PCR. For comparison, nasopharyngeal swab samples from patients infected with influenza A or B were also tested. All samples were received from outpatients in Greece.

### Ethics statement

This study was based on routine diagnosis samples from the biobank of Eginition Hospital (Athens, Greece). All collected data were anonymized in standardized forms. Written informed consent was obtained from all individuals. For the minors, consent was obtained from parents or guardians. The study was conducted in accordance with the ethical standards noted in the 1964 Declaration of Helsinki and its later amendments. The study, which is part of DopaHypoCov—SARSCoV2 2020 Institut Pasteur International Network program, has been approved by the Administration Board of the Hellenic Pasteur Institute.

### Total RNA extraction

Nasopharyngeal swab specimens were collected in 2 ml of viral nucleic acid sample preservation fluid. Total RNA extraction was performed with the MagNA Pure LC Total Nucleic Acid Isolation Kit using the MagNa Pure LC 2.0 automated nucleic acid purifier (Roche), as recommended by the manufacturer.

### RNA quantification by reverse transcription-quantitative PCR (RT-qPCR)

Viral RNA was assessed by real-time reverse transcription (RT)-PCR, using the LightMix Modular Sarbecovirus *E*-gene Kit for quantifying the viral envelope protein (E)-encoding gene and the LightMix Modular SARS-CoV-2 (COVID19) RdRP Kit for the *RdRp* gene, as well as the LightCycler Multiplex RNA Virus Master kit (Roche), according to manufacturer instructions. Myostatin (*MSTN*) mRNA levels were quantified as a reference, using the LightMix ModularDx Kit MSTN Extraction Control kit (Roche). Positive and negative control samples were analyzed in parallel.

cDNA synthesis for cellular genes was performed using the Moloney murine leukemia virus reverse transcriptase (Promega) according to the manufacturer’s protocol, oligo d(T)18 primer (New England Biolabs) and the recombinant ribonuclease inhibitor (Takara). After reverse transcription (RT), real-time quantitative PCR (qPCR) was performed in cDNA reactions using the SYBR Green-based Luna^®^ Universal qPCR Master Mix (New England Biolabs) as well as primer pairs specific for *DDC*, *ACE2*, *dACE2*, *ISG56* and *EPO* (Table 1), previously validated [5, 12, 31–33], or the cell type marker genes *EPCAM*, *CD45*, *CD74* and *LYN* (S1 Table). The primers for *DDC* bind to exons 10 and 12, and detect the majority of the known *DDC* mRNA isoforms [8, 34]. The house-keeping gene 14-3-3-zeta polypeptide (*YWHAZ*) was used as a normalization control. We routinely included negative and positive controls in each assay, and the quality of RNA extraction was verified for all samples.

**Table 1:** Priming oligonucleotides used for RT-qPCR analysis.

Gene	Orientation	Sequence (5’—3’)	Reference
*DDC*	Forward	AGAGGGAAGGAGATGGTGGATTA	[12]
	Reverse	GGGGCTGTGCCTGTGCGT	
*ACE2*	Forward	GGACCCAGGAAATGTTCAGA	[32]
	Reverse	GGCTGCAGAAAGTGACATGA	
*dACE2*	Forward	GTGAGAGCCTTAGGTTGGATTC	[5]
	Reverse	TAAGGATCCTCCCTCCTTTGT	
*ISG56*	Forward	GGACAGGAAGCTGAAGGAG	[33]
	Reverse	AGTGGGTGTTTCCTGCAA	
*EPO*	Forward	GCCCCACCACGCCTCATCTGT	[31]
	Reverse	CTTCCAGGCATAGAAATTAAC	

### Cell and virus culture

VeroE6 and A549 epithelial cell lines were cultured in Dulbecco’s modified minimal essential medium (DMEM) (Invitrogen), supplemented with 100 U/mL penicillin, 100 μg/mL streptomycin and 10% (v/v) fetal calf serum, at 37°C, 5% CO_2_. To create 3% (v/v) oxygen tension, cells were cultured in a fully humidified incubator supplied with pure nitrogen gas to reduce oxygen as well as with 5% (v/v) CO_2_ at 37 °C (New Brunswick CO_2_ incubator Innova). SARS-CoV-2 (isolate 30–287, lineage B1), derived from a positively testing oropharyngeal swab sample of a COVID-19 patient in Alexandroupolis (Greece) [35], was propagated in VeroE6 cells. Fully confluent cells were infected and four days after inoculation, the supernatant was collected and stored at −80°C for further usage. Titration was carried-out in VeroE6 cells seeded in 96-well plates that were cultured at 37°C for 4 days, and the cytopathic effect (CPE) was monitored using inverted phase contract microscopy. TCID_50_ was determined based on the method of Reed and Muench [36].

### Infection of mammalian cells

Infections of VeroE6 cells with SARS -CoV-2 (m.o.i. of 0.1) were carried out in 6-well plates. Mock-treated cells were cultured in parallel, as control. At 24 and 48 hours post-infection, cells were lysed using NucleoZOL (Macherey-Nagel) and cell lysates were analyzed using RT-qPCR.

### Measurement of intracellular ATP level

ATP was measured using the ViaLight HS BioAssay Kit (Lonza), according to the manufacturer’s protocol, in a GloMax 20/20 single tube luminometer (Promega) for 1 s. ATP levels were normalized to total protein amount.

### Statistical analyses

The relative quantification method 2^−ΔΔCt^ [37] was used to normalize the gene expression data with respect to the reference gene and obtain fold change values of positive and negative groups, using the respective gene expression values of control samples as a calibrator. Based on the Kolmogorov-Smirnov (KS) test, we verified if data distribution was parametric or non-parametric and subsequently selected the appropriate statistical test for analysis. Comparison of gene expression between positive and control group samples was performed using the student’s t-test (unpaired). We evaluated the diagnostic accuracy of gene expression with the receiver operating characteristic (ROC) regression analysis and calculated for each gene the area under the ROC curve (AUC), as well as the cut-off value based on Youden’s index. Correlations between the mRNA levels of the genes and with the viral RNA were determined by Pearson’s or Spearman’s *correlation coefficient* (*r*), for parametric and non-parametric data, respectively. We estimated the statistical relationship between gene expression and demographic data by Pearson’s *r* for age (continuous variables) and by either the parametric student’s t-test or the non-parametric Mann-Whitney U test for sex (binary variable). All statistical analyses were performed using GraphPad Prism 6.0 (GraphPad Software, Inc.) and *p*<0.05 (two-tailed) was considered statistically significant.

## Results

### Subjects

We tested nasopharyngeal swab samples from 37 COVID-19 patients showing no or mild COVID-19 clinical symptoms, including cough, sore throat, mild fever below 38 °C and loss of smell, and 38 non-infected individuals (Table 2). The median age of COVID-19 patients was 38 years (interquartile range—IQR = 31–52), and the negative control group had median age 47 years (IQR = 34–68). In comparison, nasopharyngeal swab samples from 38 patients infected with influenza A or B were also tested, and their demographic data are shown on S2 Table.

**Table 2:** Demographic data of SARS-CoV-2 positive and negative subjects.

Characteristics		SARS-CoV-2		*p* value
		Positive	Negative	
**Total Number**		37	38	
**Median age in years (IQR)**		38 (31–52)	47 (34–68)	0.062^a^
**Age group**	**0–17**	0	4	
	**18–39**	17	7	
	**40–64**	10	13	
	**65+**	3	11	
	**NR**	7	3	
**Sex**	**Male**	14	14	0.449^b^
	**Female**	14	23	
	**NR**	9	1	

NR: Data not recorded; IQR: inter-quartile range;

^a^Mann-Whitney U test;

^b^Fisher’s exact test.

### Expression of *DDC*, *ACE2/dACE2*, *ISG56* and *EPO* genes in SARS-CoV-2-infected patients as compared to non-infected individuals

#### *DDC* mRNA expression

**Fig 1:**
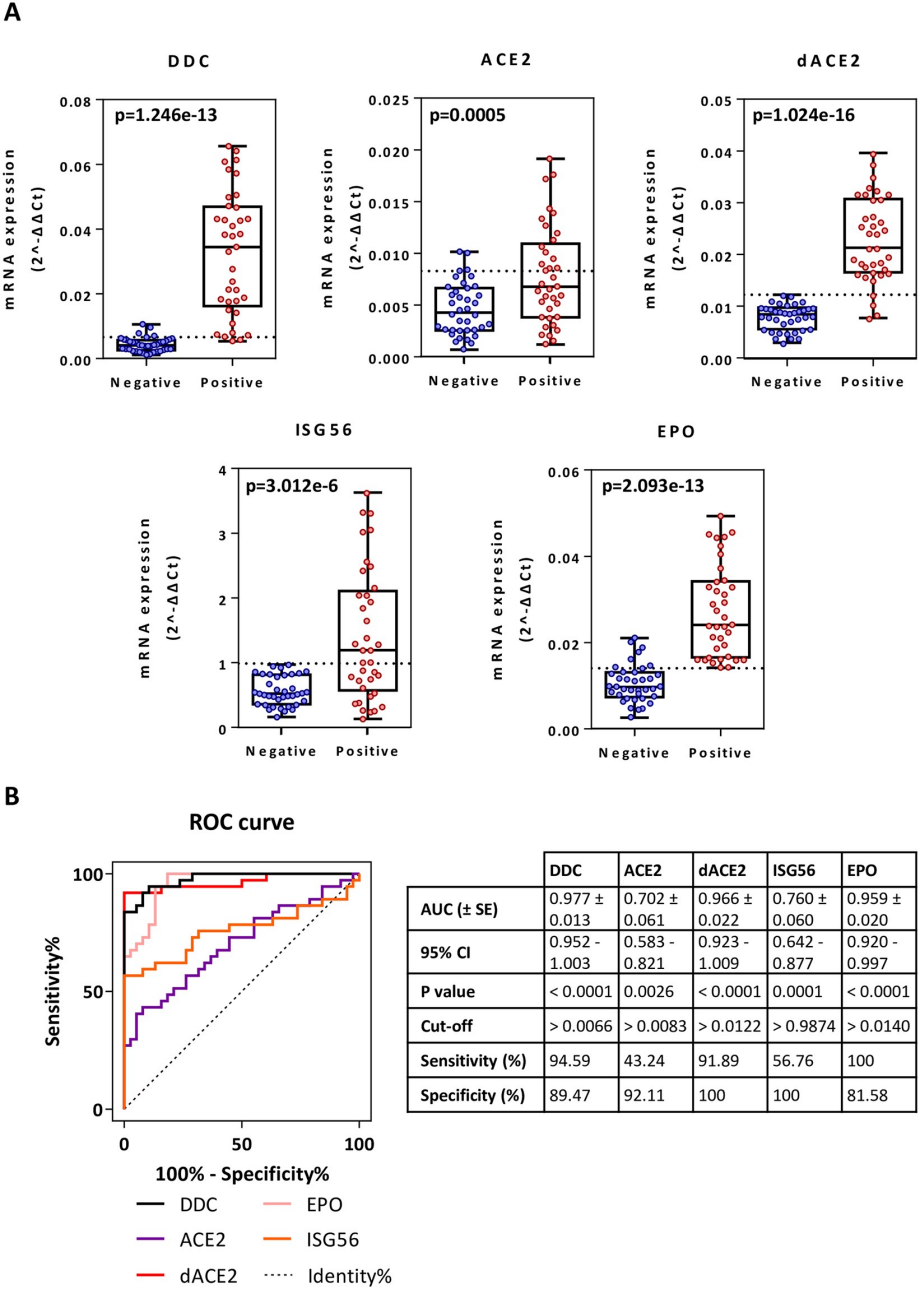
Comparison of *DDC*, *ACE2*, *dACE2*, *ISG56* and *EPO* mRNA expression in nasopharyngeal swab samples between COVID-19 patients showing no or mild symptoms and non-infected individuals (A) and ROC curve analysis (B). Relative mRNA values (mean 2^-ΔΔCt^) from the patient (n = 37) and control (n = 38) groups. The horizontal dotted line indicates the optimal threshold value (cut-off). Data are represented as box plots; line in the middle, median; box edges, 25^th^ to 75^th^ centiles; whiskers, range of values. *p* values were calculated with the student’s t-test. ROC curves were generated and AUC, 95% CI, *p* values and cut-off points with their specificity and sensitivity were calculated.

**Fig 2:**
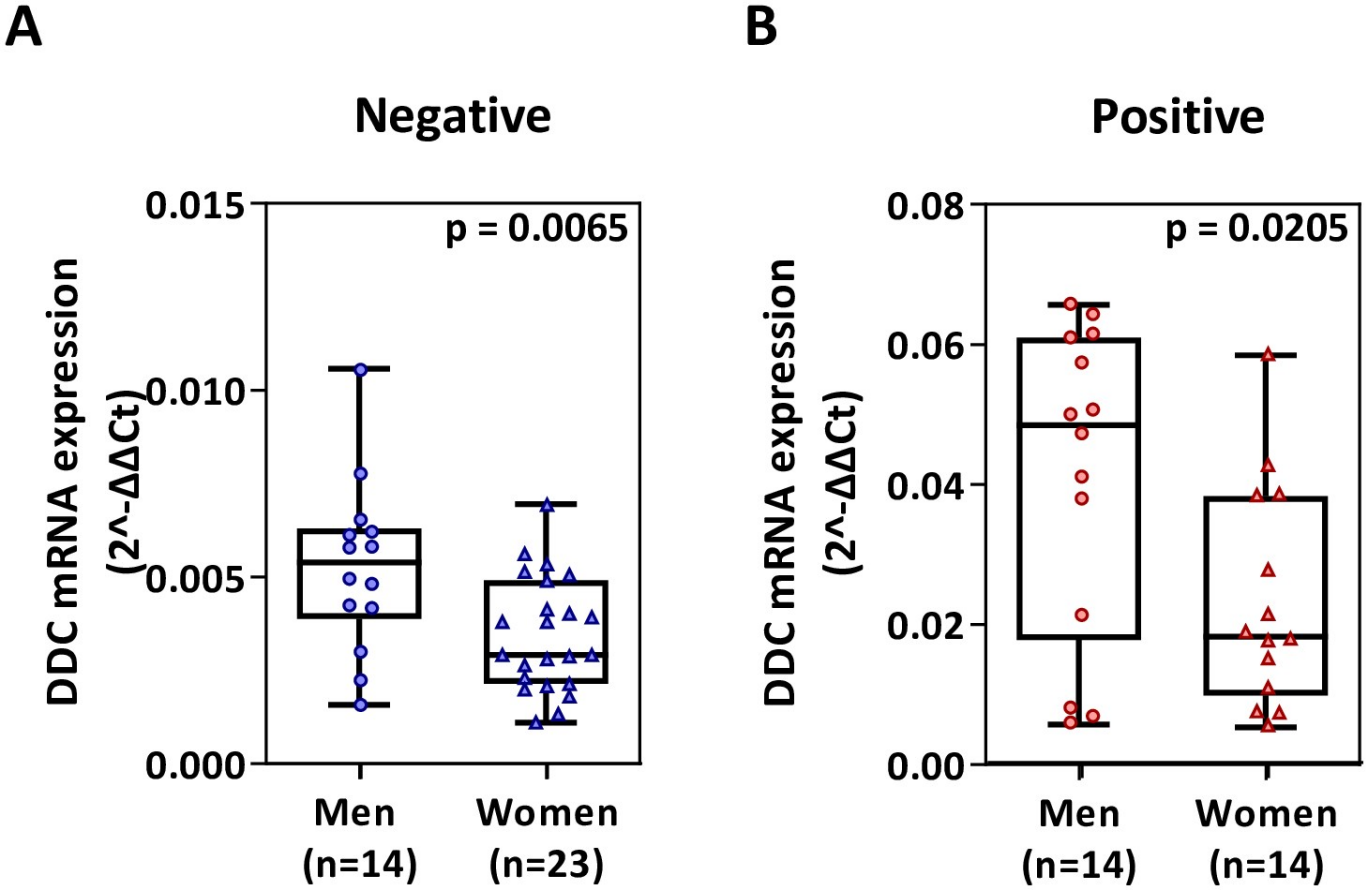
Relative *DDC* mRNA expression in men as compared to women in non-infected (A) and SARS-CoV-2-infected (B) groups. Data are represented as box plots; line in the middle, median; box edges, 25^th^ to 75^th^ centiles; whiskers, range of values. *p* values were calculated with student’s t-test.

In nasopharyngeal swab samples, a significant increase in *DDC* mRNA expression with 7.6 mean-fold (*p* = 1.2e-13) was observed in SARS-CoV-2 patients (median = 0.035; IQR = 0.016–0.047), as compared to non-infected subjects (median = 0.004; IQR = 0.003–0.006) (Fig 1A). Moreover, the ROC curve analysis revealed high ability of *DDC* to distinguish between infected and non-infected individuals (Fig 1B), with an area under the curve (AUC) of 0.98 (95% confidence interval—CI = 0.95–1.003). At the optimal cut-off value of 0.007, the sensitivity and specificity were 95% and 90%, respectively. Finally, we found that *DDC* mRNA levels were higher in men as compared to women, for both positive (1.8-fold of mean; *p* = 0.02) and negative (1.5-fold of mean; *p* = 0.007) groups (Fig 2). No correlation with age was observed (S1 Fig in S2 File).

#### *ACE2* and *dACE2* mRNA expression

The mRNA levels of the ISG *dACE2* were higher by 2.9 mean-fold (*p* = 1.02e-16) in COVID-19 patients (median = 0.021; IQR = 0.017–0.031) compared to the non-infected individuals (median = 0.008; IQR = 0.006–0.01) (Fig 1A). A lower increase of 1.7 mean-fold was detected for the viral receptor-encoding *ACE2* gene in the positive (median = 0.007; IQR = 0.004–0.011) versus the negative (median = 0.004; IQR = 0.003–0.007) group (Fig 1A). ROC curve analysis determined for *dACE2* a much higher AUC of 0.97 (95% CI = 0.92–1.01) than for *ACE2* (AUC = 0.7; 95% CI = 0.58–0.82) (Fig 1B). In addition, the cut-off point value for *dACE2* was calculated at 0.012, with a sensitivity of 92% and a specificity of 100%. No correlation was observed with sex (S2 Fig in S2 File) or age (S1 Fig in S2 File) for *ACE2* and *dACE2*.

#### *ISG56* and *EPO* mRNA expression

As *dACE2* is considered an ISG [4], we comparatively evaluated the expression of the well-studied *ISG56* gene. Similar to *dACE2*, SARS-CoV-2-infected patients had 2.5 mean-fold higher (*p* = 3.012e-6) *ISG56* mRNA levels (median = 1.194; IQR = 0.572–2.106), as compared to the non-infected subjects (median = 0.521; IQR = 0.358–0.814) (Fig 1A). However, the discriminating accuracy determined for *ISG56* (AUC = 0.76; 95% CI = 0.64–0.88) (Fig 1B) was lower than *dACE2*, with a sensitivity of 57% and a specificity of 100% at the cut-off point from the ROC (0.987).

Since *DDC* and *ACE2* expression is regulated by hypoxia [14, 17–20, 22] and the HIF-target immune-modulatory gene *EPO* [21], we concurrently examined the expression profile of *EPO* in COVID-19 patients compared to the non-infected individuals. *EPO* mRNA levels were higher by a mean-fold of 2.6 (*p* = 2.1e-13) in SARS-CoV-2 patients (median = 0.024; IQR = 0.017–0.034), as compared to the control group (median = 0.01; IQR = 0.007–0.013) (Fig 1A). Moreover, a high discriminating accuracy was determined for *EPO* (AUC = 0.96; 95% CI = 0.92–1.0) (Fig 1B). At the cut-off point from the ROC (0.014), *EPO* showed a sensitivity of 100% and a specificity of 82%.

### In nasopharyngeal swabs, the expression of *DDC* is specifically altered in SARS-CoV-2-infected patients

**Fig 3:**
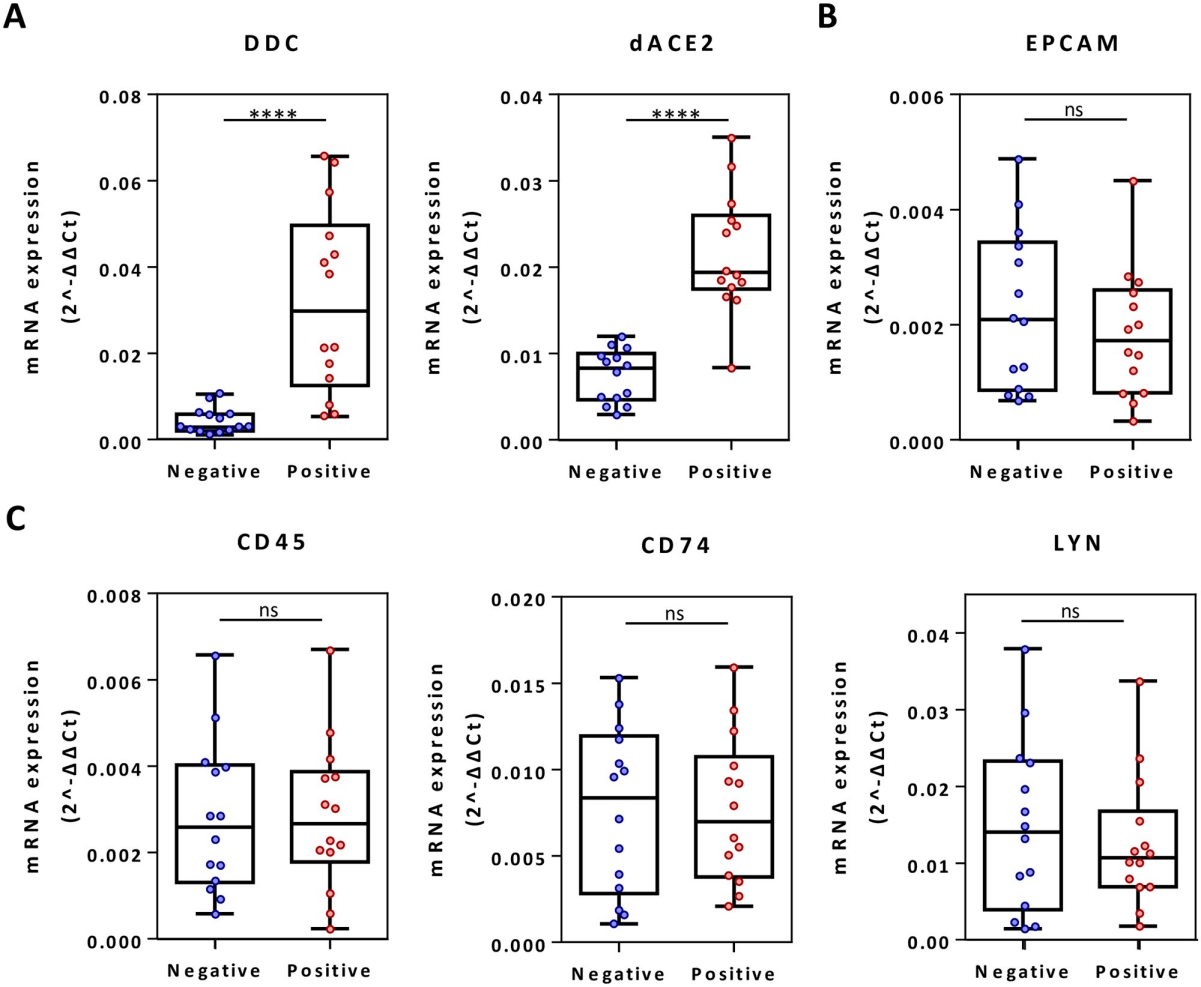
Comparison of the mRNA expression of *DDC* and *dACE2* (A), epithelial marker *EPCAM* (B) and immune cell markers *CD45*, *CD74* and *LYN* (C) between COVID-19 patients and non-infected individuals. Data are represented as box plots; line in the middle, median; box edges, 25^th^ to 75^th^ centiles; whiskers, range of values. *p* values were calculated with student’s t-test. ****:p<0.0001, ns: Non-significant.

We sought to exclude the possibility that the detected alterations of *DDC* and *dACE2* expression in SARS-CoV-2 infected patients might be due to enrichment of specific inflammation-related cell populations expressing these genes in nasopharyngeal tissue. For this, we compared gene expression of the epithelial cell marker *EPCAM* and immune cell markers *CD45*, *CD74* and *LYN* (for macrophages, neutrophils, dendritic, natural killer, B and T lymphocytes) between COVID-19 positive and negative sample subsets representing the entire range of *DDC* and *dACE2* expression (Fig 3A). Both epithelial (Fig 3B) and immune (Fig 3C) cell markers had no significant differences between the two groups, possibly because the COVID-19 samples are derived from patients with only mild or no symptoms. To validate the primers used in the analysis, we compared the expression of these markers in airway epithelial A549 and monocytic THP-1 cell lines (S3 Fig in S2 File).

Next, the specific cell types expressing *DDC* in the SARS-CoV-2-infected nasopharyngeal tissue were determined by analyzing the gene expression data of the previously reported single-cell RNA-Sequencing study [6] that are available in Magellan COVID-19 data explorer (https://digital.bihealth.org). According to the dataset derived from 14 nasopharyngeal swab samples, *DDC* is almost exclusively expressed in secretory and ciliated epithelial cells of COVID-19 patients and not in immune cells (S4 Fig in S2 File).

**Fig 4:**
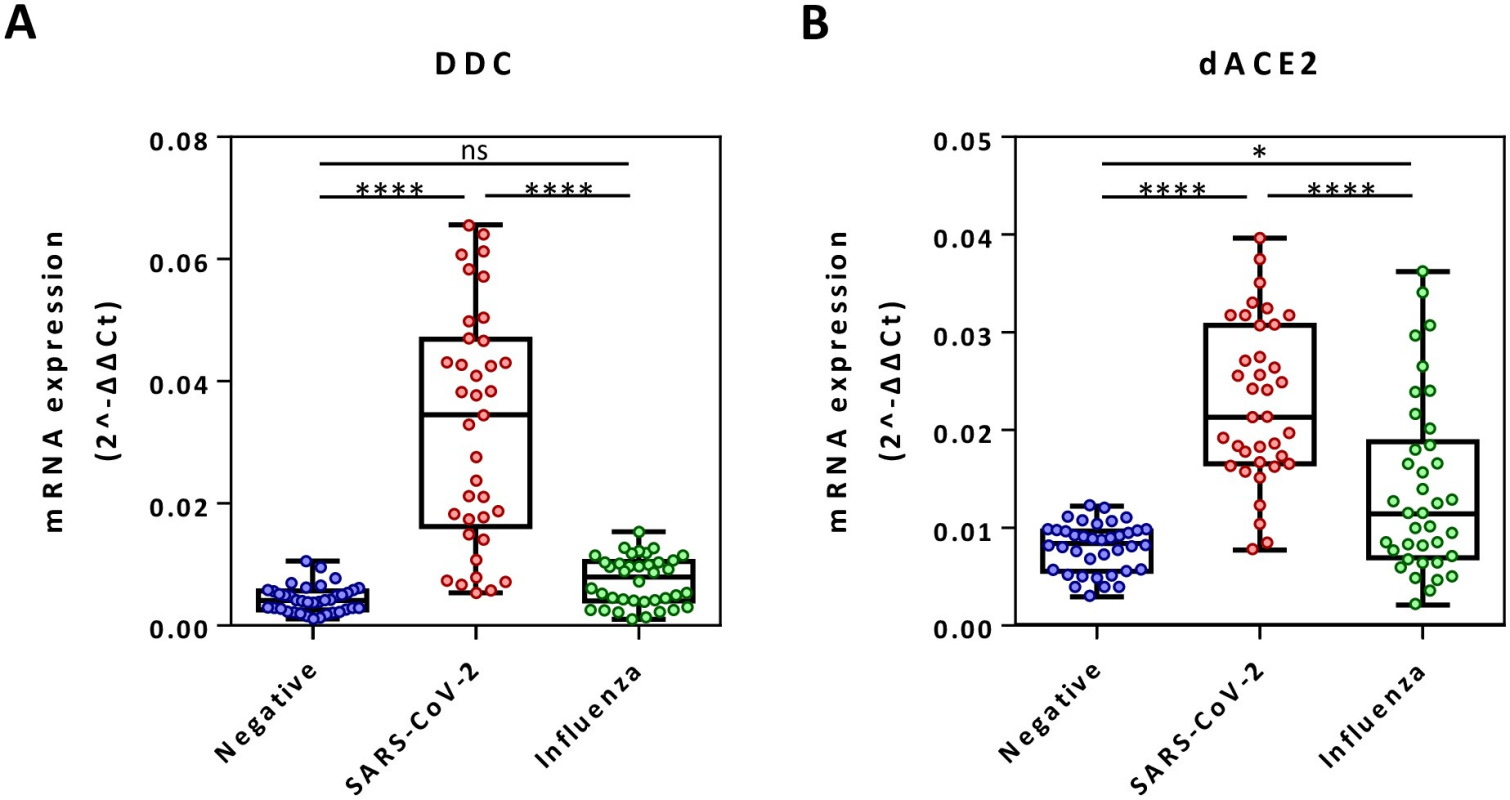
Comparing *DDC* and *dACE2* mRNA levels of influenza A/B-positive nasopharyngeal swab samples with the ones from SARS-CoV-2 positive and negative samples. mRNA values (mean 2^-ΔΔCt^) from the influenza-infected group (n = 38) compared to the ones from the SARS-CoV-2 (n = 37) and negative control (n = 38) groups of Fig 1. Data are represented as box plots; line in the middle, median; box edges, 25^th^ to 75^th^ centiles; whiskers, range of values. *p* values were calculated with Kruskal-Wallis test.

To elucidate if *DDC* expression is altered specifically in SARS-CoV-2 infection or also in other respiratory viral infections, influenza-positive swab samples were analyzed in comparison to the COVID-19 and infection-negative ones presented above. In contrast to COVID-19, *DDC* mRNA levels in the influenza-positive samples were not significantly different than the ones in the negative control group (Fig 4A). For *dACE2*, a slightly higher expression by 1.8 mean-fold (*p* = 0.0125) was shown in influenza-positive (median = 0.008; IQR = 0.004–0.010) versus negative samples (Fig 4B), which is however lower than the one observed in the SARS-CoV-2 group (2.9 mean-fold).

### Correlation analysis between SARS-CoV-2 viral load and gene expression of *DDC* and ACE2/*dACE2*

**Fig 5:**
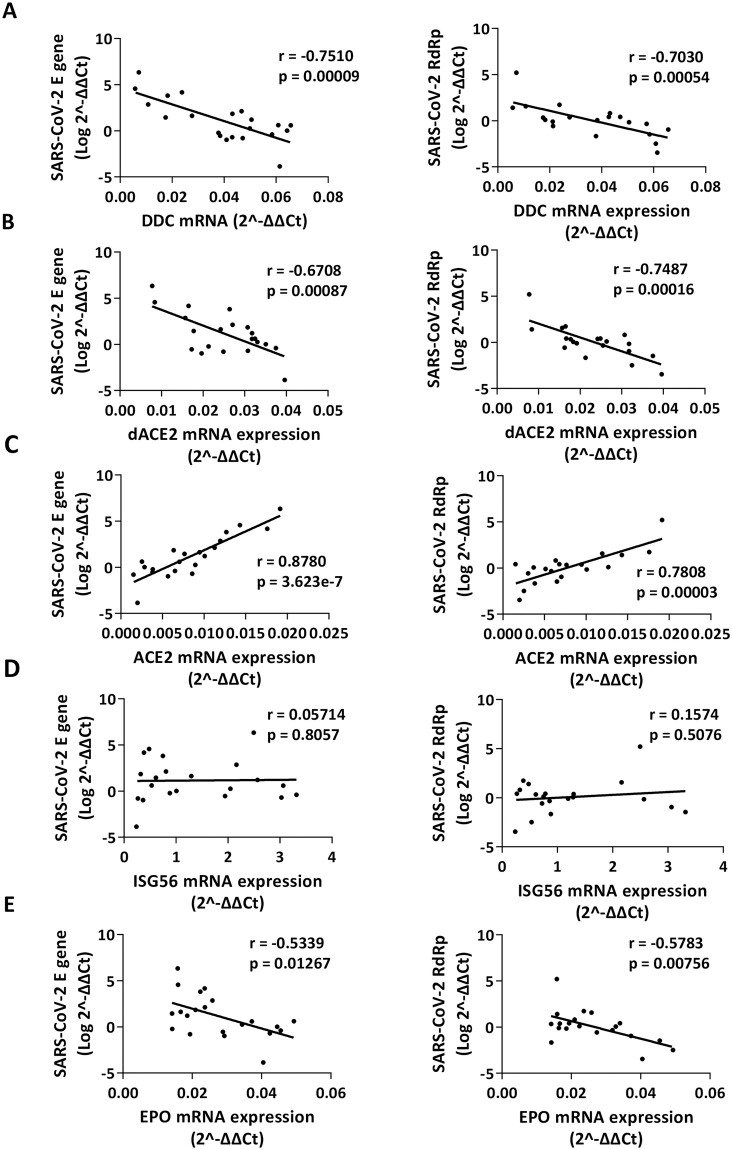
XY scatter plot and fitted linear regression lines of SARS-CoV-2 *E* (left) and *RdRp* (right) RNA levels versus *DDC* (A), *dACE2* (B), *ACE2* (C), *ISG56* (D) and *EPO* (E) mRNA expression. Viral RNA levels were determined using primers specific for E (n = 21) and/or RdRp (n = 20) coding regions. Viral load was log transformed (base 10) prior to calculation of the Pearson’s correlation coefficient (*r*) and *p* values (*p*).

Because the expression of *DDC* and *ACE2*/*dACE2* genes was significantly higher in SARS-CoV-2 infected patients, we further assessed the relationship between the respective gene mRNA levels and viral load, as measured by the detection of the viral RNA levels. Interestingly, while viral RNA amounts were positively associated with *ACE2* expression (Pearson’s r ≥0.8, p <0.001), they had a strong negative correlation (r ≤−0.7, *p* <0.001) with *DDC* and *dACE2* mRNA levels (Fig 5A–5C), with similar results for both *E* and *RdRp* regions. Moreover, viral RNA had no correlation with *ISG56* and a moderate negative correlation with *EPO* expression (r ≤−0.5, p <0.05) (Fig 5D and 5E).

### Correlation of the mRNA levels of *DDC* with *dACE2*

**Fig 6:**
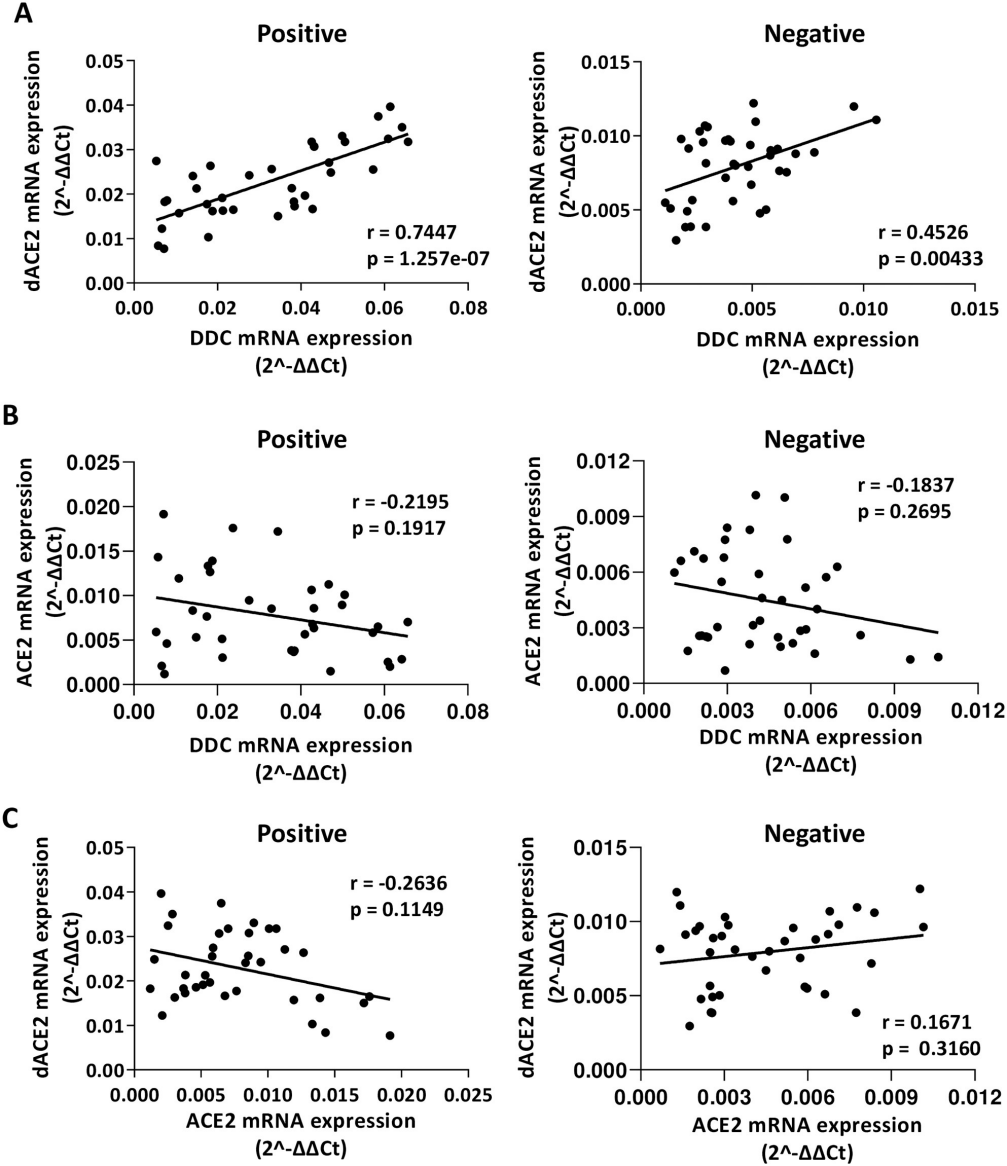
Correlation among *DDC*, *dACE2* and *ACE2* gene expression levels in SARS-CoV-2-positive and negative nasopharyngeal swab samples. XY scatter plot and fitted linear regression lines of the mRNA levels of (**A**) *DDC* versus *dACE2*, (**B**) *DDC* versus *ACE2* and (**C**) *dACE2* versus *ACE2*, in positive (left) and negative (right) samples. Pearson’s or Spearman’s correlation coefficient (*r*) and *p* values (*p*) were calculated.

**Fig 7:**
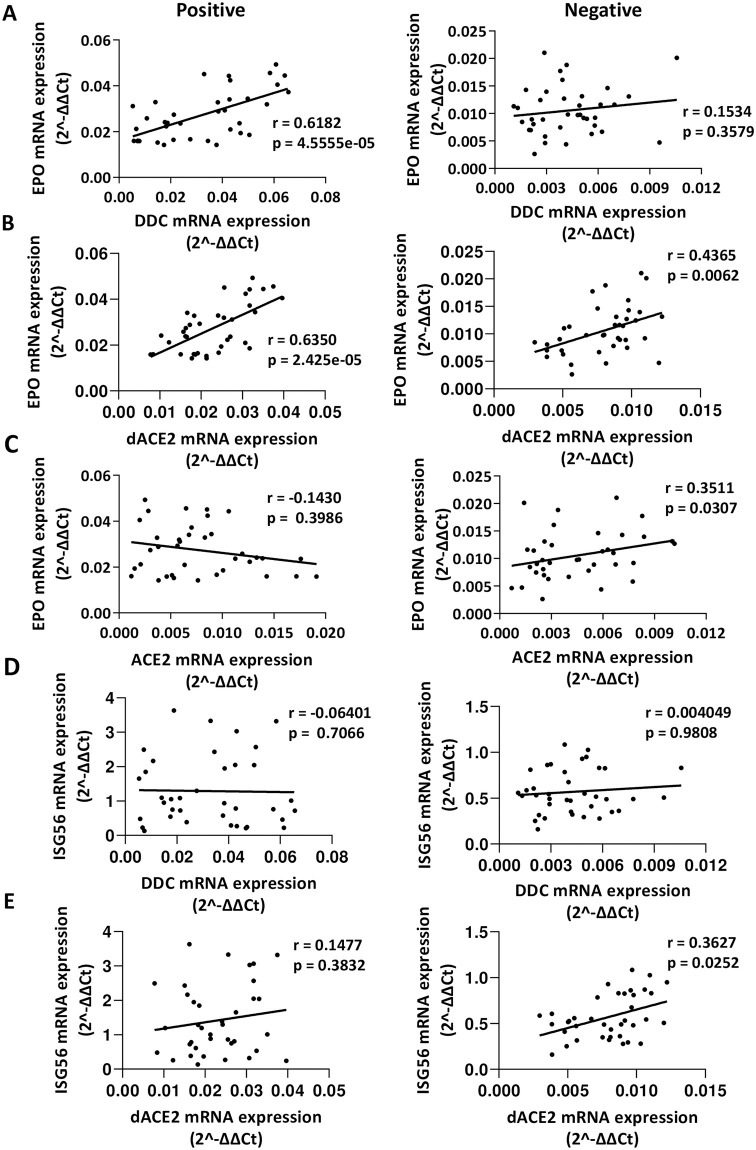
Correlation of *EPO* and *ISG56* with *DDC*, *dACE2 and ACE2* gene expression levels in SARS-CoV-2-positive and negative nasopharyngeal swab samples. XY scatter plot and fitted linear regression lines of the mRNA levels of (**A**) *DDC* versus *EPO*, (**B**) *EPO* versus *dACE2*, (**C**) *EPO* versus *ACE2*, (**D**) *ISG56* versus DDC, and (**E**) ISG56 versus *dACE2*, in positive (left) and negative (right) samples. Pearson’s or Spearman’s correlation coefficient (*r*) and *p* values (*p*) were calculated.

Next, we examined the association of mRNA expression among the genes studied in COVID-19 patients and non-infected subjects. In the virus-infected group, we observed a strong correlation between *DDC* and *dACE2* mRNA levels (Pearson’s *r* = 0.74, *p* = 1.3e-07, Fig 6A), while a less robust correlation (*r* = 0.45, *p* = 0.004, Fig 6A) was identified in the non-infected group. However, no such correlation was found between the expression of *ACE2* and that of *DDC* or *dACE2* (Fig 6B and 6C). In addition, moderate correlations were observed between the expression of *EPO* with that of *DDC* (*r* = 0.62, *p* = 4.6e-05, Fig 7A) or *dACE2* (*r* = 0.64, *p* = 2.5e-05, Fig 7B) in the virus-infected individuals. The non-infected group presented significant but less robust correlations of *dACE2* with *EPO* mRNA levels (*r* = 0.44, *p* = 0.006, Fig 7B) and no correlation of *DDC* with *EPO* (*p* = 0.36, Fig 7A). No correlation was shown between the expression of *EPO* with that of *ACE2* (Fig 7C) and of *ISG56* with *DDC* or with *ACE2*/*dACE2* (Fig 7D and 7E, S5 Fig in S2 File). These results suggested that a statistically significant correlation between *DDC* and *dACE2* expression exist in SARS-CoV-2 nasopharyngeal samples. Similarly, strong correlation was also found in whole blood samples of both SARS-CoV2-infected (*r* = 0.70, *p* = 0.002) and non-infected (*r* = 0.69, *p* = 0.003) individuals (S6 Fig in S2 File).

### Sars-CoV-2 downregulates *DDC*, *dACE2* and *EPO* in cultured cells

**Fig 8:**
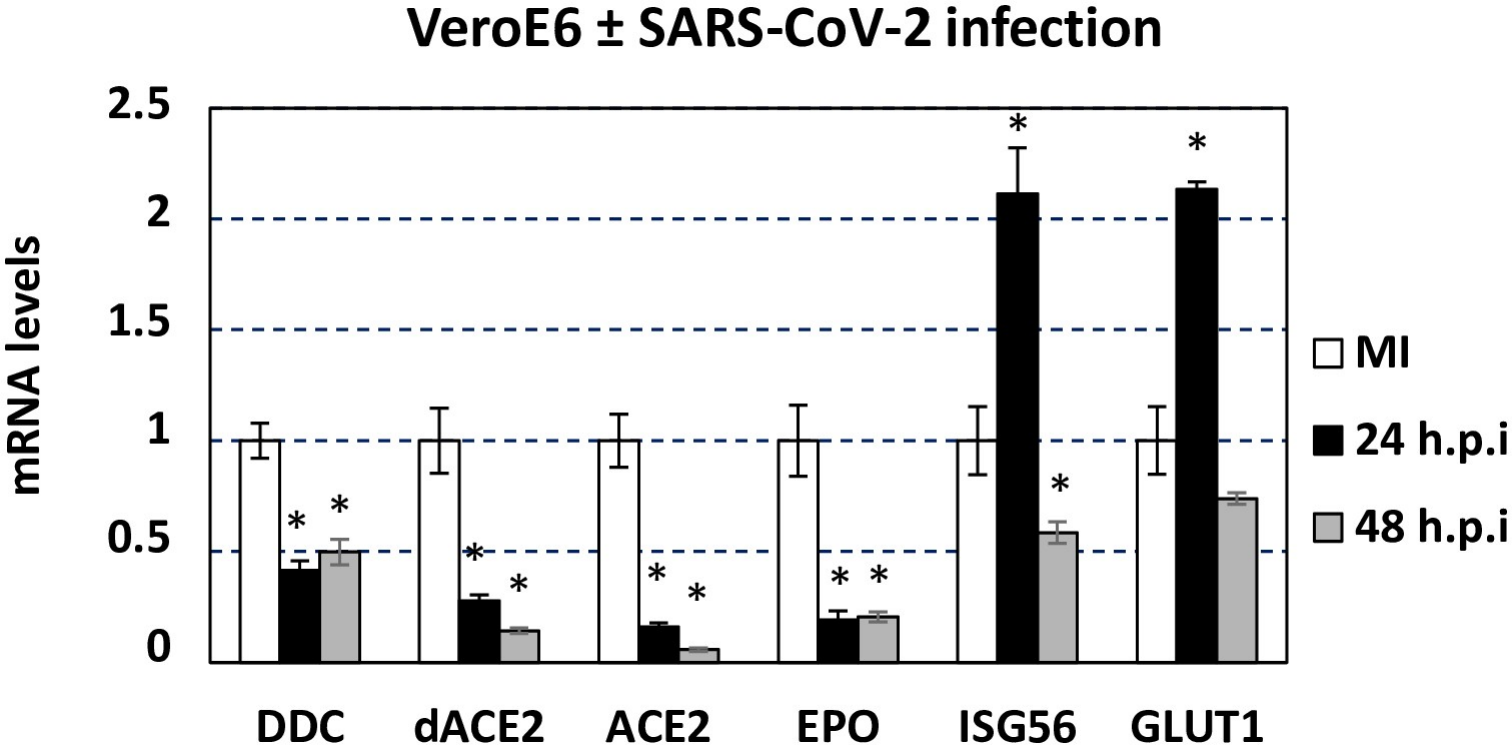
Effect of SARS-CoV2 infection in *DDC*, *dACE2*, *ACE2*, *EPO* and *ISG56* gene expression *in vitro*. VeroE6 cells were inoculated with the SARS -CoV-2 isolate 30–287 of lineage B1. Mock-infected (MI) cells were cultured in parallel, as control. 24 and 48 hours post-infection cells were lysed and cell lysates were analyzed using RT-qPCR. YWHAZ mRNA levels were used for normalization. Bars represent mean values from three independent experiments in triplicates. Error bars indicate standard deviations. *p<0.001 vs MI cells (Student’s t test).

As the expression of *DDC* and *dACE2* was negatively correlated with the viral load in SARS-CoV2 infected patients, we aimed to directly verify if the virus downregulates these genes in the target epithelial cells. For this, VeroE6 cells were infected with a SARS-CoV-2 strain of lineage B1 for 24 or 48 hours. Upon viral infection, there was a substantial reduction in the mRNA levels of *DDC* (2-fold) and *dACE2* (3.5- and 7-fold, at 24 h and 48 h post-infection, respectively), as well as in the *ACE2* mRNA levels (6.3- and 17.5-fold at the two time points tested) (Fig 8). A different kinetics was noticed for *ISG56* mRNA, which showed an about 2-fold increase at 24 h post-infection but afterwards was suppressed by 1.7-fold. Similar to *DDC* and *dACE2*, the virus negatively affected *EPO* expression by about 5-fold. On the other hand, expression analysis of another hypoxia marker gene *GLUT1* showed an upregulation at 24 h post-infection. In accordance to these results in VeroE6, processing of data acquired from the RNA-sequencing Skyline database (http://rstats.immgen.org/Skyline_COVID-19/skyline.html) [38] revealed that SARS-CoV-2 infection reduced the expression of *DDC* also in A549, A549 overexpressing ACE2 and Calu3 cell lines (S7A Fig in S2 File) and that of *EPO* in A549 (S7B Fig in S2 File), whereas increased other hypoxia markers, such as *GLUT1* and *VEGFA* (S7C and S7D Fig in S2 File).

**Fig 9:**
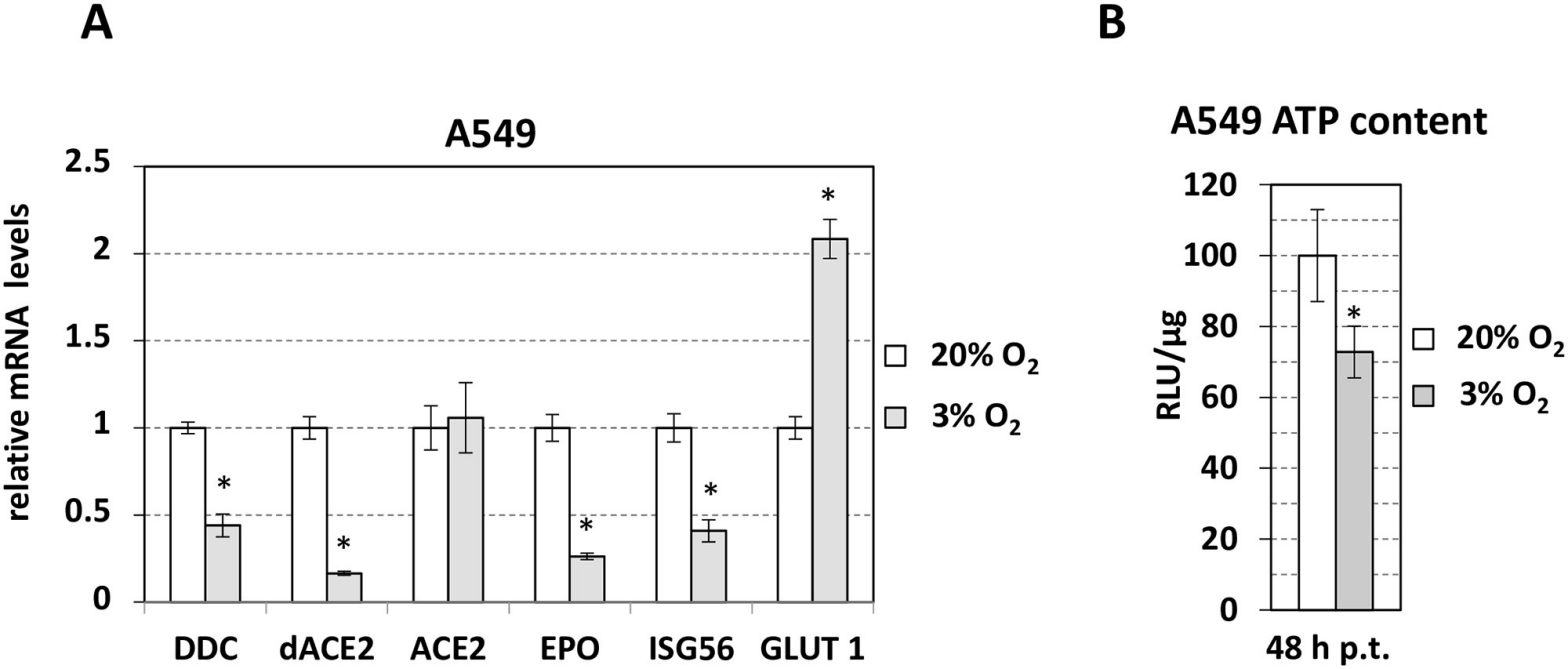
Effect of hypoxia on *DDC*, *dACE2*, *ACE2*, *ISG56*, *EPO* and *GLUT1* expression and on cell viability in A549 lung epithelial cells. Cells were cultured under atmospheric (20% v/v) or hypoxic (3% v/v) O_2_ tension for 48 h. (**A**) Cell lysates were analyzed using RT-qPCR. YWHAZ mRNA levels were used for normalization. Bars represent mean values from three independent experiments in triplicates. Error bars indicate standard deviations. *p<0.001 vs 20%-cultured cells (Student’s t test). (**B**) Bioluminescent measurement of intracellular ATP levels. Mean values (%) were normalized to ATP levels detected in cells cultured at 20% O_2_. *p<0.01 vs 20%-cultured cells (Student’s t test). h p.t: hours post-treatment at 20 or 3% O_2_.

The upregulation of *GLUT1* and *VEGFA* in SARS-CoV-2-infected cells suggest the activation of a hypoxia-associated signaling upon viral infection. On the other hand, the negative effect in *EPO* expression may be exerted by the virus in an attempt to reduce the IFN-related [39, 40] immune-modulatory function of erythropoietin [41]. Another reason for SARS-CoV-2 to target *EPO* could be the anti-apoptotic properties [42] of the encoded protein, as the virus induces cell death in VeroE6, shown in S8 Fig in S2 File [35] and also in previous studies [43, 44]. To investigate the possibility that the virus-mediated effects on the expression of the genes under study in cell culture are related to hypoxia and/or cell viability, we analyzed their expression in airway epithelial cells under low oxygen tension as well as in a number of conditions that promote or not cell death. To simulate the effect of hypoxia in A549 cells, we cultured them under 3% O_2_ for 48 hours compared to atmospheric O_2_ levels (20% O_2_) that these cells physiologically sense *in vivo*. The expression of *GLUT1* gene was analyzed as a marker of hypoxia. As shown in Fig 9A, *GLUT1* expression was found increased at 3% O_2_ in A549, verifying that these cells had activated the hypoxia-related cellular response. *DDC*, *dACE2*, *EPO* and *ISG56* expression was significantly reduced at 3% O_2_, whereas no effect was exerted by hypoxia on *ACE2* expression. This downregulation coincided well with a hypoxia-mediated 25% reduction in cell viability of A549 (Fig 9B). These results are in agreement with previous data showing that prolonged hypoxia (≥12 h) triggers apoptosis in these cells [45] and destabilizes HIF-1α mRNA, causing a reduction in the expression of its target genes (e.g. *EPO*), in contrast to HIF-2α that is increased in both acute and prolonged hypoxia and regulates *GLUT1* and *VEGFA* [26]. In contrast to A549 cells, hepatic epithelial cells (Huh7.5) showed increased *EPO* expression after long exposure to 3% O_2_ (S9A Fig in S2 File), a condition previously shown to positively affect the viability of these cells [46].

To further explore the link of the reduction in *DDC*, *dACE2* and *EPO* expression with virus-induced cell death, we comparatively employed the infection models of DENV and HCV at late hours post-infection. DENV promotes cell death [47], as does SARS-CoV2, whereas HCV does not affect cell viability of Huh7.5 cells [46]. As shown in S9B and S9C Fig in S2 File, DENV reduced the expression of *EPO*, similar to SARS-CoV2 (Fig 8), while no effect was exerted by HCV at the late time-point tested. In addition to *EPO*, *DDC* [14] and *dACE2* were also suppressed by DENV, but *dACE2* was not suppressed by HCV. On the other hand, both viruses positively affected the expression of *ACE2*, which is in accordance with previous data supporting *ACE2* upregulation upon DENV infection [48] and a negative correlation between the expression of *ACE2* and many interferon-stimulated genes [49].

## Discussion

Our results showed that the gene expression of *DDC* (L-Dopa decarboxylase) [8] and of the recently reported interferon-inducible truncated *dACE2* isoform [4, 5] was significantly higher in nasopharyngeal swab samples from COVID-19 patients with no or mild symptoms, as compared to non-infected individuals, while it was negatively correlated with SARS-CoV-2 RNA levels in patients and was negatively affected by the virus in cell culture-infected epithelial cells. A similar pattern of expression to *DDC* and *dACE2* was also observed for the hypoxia-regulated immune-modulatory gene *EPO*, in SARS-CoV-2-infected individuals and cultured cells. The detected differences in gene expression in the COVID-19 nasopharyngeal samples were not resulted from an enrichment of specific cell populations in this tissue, as there was no significant difference in the expression of epithelial and immune cell marker genes between SARS-CoV-2 positive and negative samples. *DDC* expression did not differ between influenza-positive and negative swab samples, underlining the specific relation of *DDC* regulation with SARS-CoV-2 infection. Moreover, the negative correlation between the mRNA levels of *DDC*, *dACE2* and *EPO* with viral load in SARS-CoV-2 positive samples combined with their downregulation in infected VeroE6 cells, that mount an inefficient innate response [50, 51], suggest that SARS-CoV-2 genome replication and virus spread occurs in parallel with the suppression of these genes. Based on the above, we assume that the expression reprogramming of *DDC*, *dACE2* and *EPO* in COVID-19 patients, as compared to non-infected individuals, possibly constitute an antiviral immune strategy of the infected host. Alternatively, SARS-CoV-2 might benefit from the lower levels of expression of these genes in certain individuals to achieve efficient propagation. On the other hand, the viral receptor encoding mRNA *ACE2* exhibited a lower increase in SARS-CoV-2 positive samples and a similar expression pattern in VeroE6 infected cells as compared to *dACE2*, whereas its expression levels positively correlated with the viral load in patients. This possibly suggests that the increased expression of *ACE2* in certain individuals makes them vulnerable to SARS-CoV-2 infection resulting in high virus titers. A positive effect from SARS-CoV-2 on *ACE2* is less possible, as the virus was shown to downregulate *ACE2* in our *in vitro* studies. A similar increase to *dACE2*, was also observed for another interferon-stimulated gene, *ISG56*, in COVID-19 patients but no correlation was shown with viral load. Furthermore, we revealed a significant correlation in the expression levels of *DDC* and *dACE2*, whereas no correlation was found with *ACE2* in nasopharyngeal swab and blood samples of both SARS-CoV-2-infected and non-infected individuals.

Concerning *DDC*, this is the first report on the involvement of an enzyme gene belonging to the catecholamine/serotonin synthesis pathway in SARS-CoV-2 infection. Processing of the single cell gene expression data (RNA-sequencing) of Chua et al. [6] derived from nasopharyngeal swabs of COVID-19 patients revealed that *DDC* is almost exclusively expressed in epithelial cells (secretory, ciliated and FOXN4) and not in immune cell populations. This is in agreement with the absence of an effect on *DDC* expression in other viral diseases causing inflammation of the respiratory tract, such as influenza A or B, and as mentioned above, this underlines the specific association of SARS-CoV-2 infection with *DDC* regulation. Our *in vitro* results showing the downregulation of *DDC* after SARS-CoV-2 infection of VeroE6 cells are consistent with other *in vitro* data in A549 and Calu3 lung epithelial cells (S7 Fig in S2 File [38]). Interestingly, to our knowledge SARS-CoV-2 is the third virus found to cause DDC downregulation [14]. In relation to the present finding of *DDC* and SARS-CoV2 negative association in the nasopharyngeal swabs, our previous data have shown the negative correlation of *DDC* mRNA levels with HCV RNA in patient liver samples, and the downregulation of DDC mRNA and protein by HCV and DENV replication in cultured cells of non-neuronal origin [14]. A negative relationship between SARS-CoV-2 and this pathway is further supported by a study showing that the catecholamine degradation enzyme COMT is required for SARS-CoV-2 infection [16]. Moreover, interactome and proteome analyses revealed the association of the viral proteins ORF7b, NSP4, NSP7 and M with COMT, as well as of ORF7b with the catecholamine-catabolizing enzyme MAOA [15, 52]. Effects caused by *DDC* expression and DDC-activity related products on SARS-CoV-2 proliferation are also possible, as our previous experiments have shown that HCV and DENV RNA replication was reduced upon DDC overexpression and treatment with dopamine or serotonin, while it was favored upon DDC inhibition [14]. In addition, activation of serotonin receptor signaling by agonist 5-nonyloxytryptamine has been found to diminish infectivity of the coronavirus mouse hepatitis virus, the reovirus and the alphavirus chikungunya [53]. According to the Chinese academy of science, series of tryptamine derivatives have anti-viral activity against hepatitis B virus replication [54]. In turn, yellow fever and Coxsackie B4 viral infections impair persistently the synthesis of catecholamines (dopamine, noradrenaline) in infant mice [55]. The reduction of *DDC* expression by SARS-CoV2, which results in hampered biosynthesis of the neurotransmitters dopamine and serotonin, provides insight in the cause of the neurological manifestations frequently observed in these patients. In line with this, previous data on the crucial role of DDC-dependent synthesis of serotonin in taste bud cells for normal taste functions [56] might explain the high incidence of smell and/or taste loss in patients [57]. Moreover, serotonin has been shown to exert functions in innate as well as adaptive immunity, [58] thus a putative SARS-CoV-2-mediated deregulation of its production may be extremely beneficial for the virus.

An upregulation in SARS-CoV-2-infected patients, as compared to the control group, and a negative correlation with viral RNA levels was also observed for *dACE2* transcript. Our data on the expression pattern of *dACE2* in the nasopharyngeal tissue and in *in vitro* studies in VeroE6 cells are possibly explained by the reported function of *dACE2* as an ISG [4, 5] and the suppression of interferon signaling by SARS-CoV-2 [38], combined with the insufficient production of IFNs in response to viral infection in VeroE6 [50, 51]. Indeed, Onabajo O. et al. have previously shown that *dACE2* is not inducible by IFNs in VeroE6 [4]. As SARS-CoV-2 is known to be highly potent in inhibiting IFN expression and signaling from the type I IFN receptor, compared to other respiratory viruses [39, 40], similar to our data, in infected cultures of bronchial epithelial cells no significant induction of IFN-stimulated genes has been observed [4]. Similar to SARS-CoV-2, another RNA virus that efficiently inhibits IFN response, DENV [59], also reduces both *dACE2* and *DDC*. Based on single cell RNA-seq analyses (further analysis of Chua et al. data that are available in https://digital.bihealth.org; and [60]), the common chromosomal locus of *ACE2*/*dACE2* was found to be transcribed in epithelial cell subpopulations of the nasal airway tissue, where innate immune genes are highly expressed [60]. Thus, combined our findings on *dACE2* and *ACE2* support the recently reported hypothesis that the 3-fold higher *ACE2* levels shown in a transcriptome analysis of nasopharyngeal COVID-19 patient samples, compared to the control group [6], are due to *dACE2* and not *ACE2* [4].

A similar increase to *dACE2*, we also detected for another interferon-stimulated gene, *ISG56*, in COVID-19 patients as compared to the non-infected individuals, in agreement with the study of Scagnolari C. et al. on oropharyngeal swabs [61], as well as an *in vitro* transient, slight upregulation early after SARS-CoV2 infection and subsequent decrease. However, in contrast to *dACE2*, no correlation was shown in patients between *ISG56* mRNA levels and viral load, which implies that these two ISGs are subjected to different regulation mechanisms during viral infection.

Compared to *dACE2* and *ISG56*, *EPO* exhibited a similar induction in response to SARS-CoV-2 infection in patients but had a moderate negative correlation with viral load. In cell culture, *EPO* expression was reduced in SARS-CoV-2 infected VeroE6 cells, consistently with the RNA-seq data of Blanco-Melo et al. in A549 cells (S7 Fig in S2 File; [38]), in contrast to other hypoxia markers (*GLUT1*, *VEGFA*) that were increased upon viral infection. The later indicates that SARS-CoV-2 triggers a hypoxia-like response in infected cells. On the other hand, the SARS-CoV-2 infection-mediated effect on *EPO*, in VeroE6 cells, is possibly related with the induction of cell death. Indeed, *EPO* expression was reduced in hypoxic A549 cells, in agreement with previous reports showing that long exposure to hypoxia (≥12 h) triggers apoptosis in A549 [45] and destabilizes HIF-1α mRNA, causing a reduction in the expression of its target genes (e.g. *EPO*), but not in HIF-2α targets (*GLUT1* and *VEGFA*) [26]. *EPO* was also suppressed by the apoptotic virus DENV in hepatic epithelial cells, but not after hypoxia treatment, a condition that increases viability of these cells [46]. Overall these results are in line with the proposed use of human recombinant EPO in severe COVID-19 cases as beneficial for the alleviation of respiratory inflammation [62]. Furthermore, our experiment in A549 cells showed that hypoxia is a condition that downregulates all three genes *DDC*, *dACE2* and *EPO*, whereas has no impact on *ACE2* expression, suggesting that hypoxia signaling in these cells may constitute a possible mechanism of co-regulation of the three genes during SARS-CoV-2 infection.

Finally, we showed that the statistically significant expression correlation of *DDC* with *dACE2* and of both *DDC* and *dACE2* with *EPO* in nasopharyngeal tissue was higher in SARS-CoV-2-infected as compared to the non-infected individuals, while no correlation with the expression of the viral receptor gene *ACE2* was found. These data complements previous ones on the co-expression of *DDC* with total *ACE2* in epithelial cells of other tissues [7] and on the regulation of *DDC* gene expression by HIF [14, 17] and EPO [21]. *DDC*, *dACE2* and *EPO* mRNA levels correlated well also in whole blood of SARS-CoV-2-infected as well of non-infected individuals. This tissue is a significant source of RNAs derived from vesicles and ribonucleoproteins secreted from different cells and tissues of the body [63].

## Conclusion

This study, combining data derived from COVID-19 patients and cultured SARS-CoV2-infected cells, provides new knowledge on the involvement of the dopamine biosynthetic enzyme gene *DDC* in SARS-CoV-2 infection and its association with *dACE2* expression, which could lead to further understanding of COVID-19 pathogenesis. Our results support that, *DDC* and *dACE2* are upregulated in patients with mild or no symptoms as a response to SARS-CoV-2 infection, while there is a negative association between their expression and viral RNA levels and *in vitro* the virus acts to suppress their expression. Thus, their reprogramming is possibly related with an antiviral response of the infected individuals, as the virus downregulates these genes to favor its propagation. Moreover, the finding that the mRNA levels of *DDC* and *dACE2* correlate well between them in COVID-19 nasopharyngeal and whole blood samples, but not with the viral receptor encoding isoform *ACE2*, complements and extends previous studies on the co-expression link of *DDC* with *ACE2* locus in epithelial cells of other tissues. Finally, we presented data showing that, in airway epithelial cells, SARS-CoV-2 infection triggers a hypoxia-like condition, and interestingly hypoxia reduces cell viability and suppresses *DDC*, *dACE2* and the HIF-target gene *EPO* in these cells. Further investigation is needed to address the involvement of these genes in disease severity.

## Supporting information

S1 Table Priming oligonucleotides used for RT-qPCR analysis of cell type marker genes. (DOCX)

S2 Table Demographic data of influenza positive and negative subjects. (DOCX)

S1 File Supplementary experimental procedures. (DOCX)

S2 File (DOCX)
