# Supplementary material for: Alteration of L-Dopa decarboxylase expression in SARS-CoV-2 infection and its association with the interferon-inducible ACE2 isoform
Source: PLoS One. 2021 Jun 29;16(6):e0253458. doi: 10.1371/journal.pone.0253458 (PMC8241096; doi:10.1371/journal.pone.0253458)
Supplement: S1 Table — (DOCX) [file pone.0253458.s001.docx]

**S1 Table:** **Priming oligonucleotides used for RT-qPCR analysis** **of** **cell type marker genes**

| Gene | Orientation | Sequence (5’ - 3’) | Reference |
| --- | --- | --- | --- |
| *EPCAM* | Forward | CGCAGCTCAGGAAGAATGTG | [1] |
|  | Reverse | TGAAGTACACTGGCATTGACG |  |
| *CD45* | Forward | AAAAGTGCTCCTCCAAGCCA | [2] |
|  | Reverse | TGG GAG GCC TAC ACT TGA CA |  |
| *CD74* | Forward | CCGGCTGGACAAACTGACA | https://pga.mgh.harvard.edu/primerbank |
|  | Reverse | GGTGCATCACATGGTCCTCTG |  |
| *LYN* | Forward | TTCTGGTCTCCGAGTCACTCA | https://pga.mgh.harvard.edu/primerbank |
|  | Reverse | GCCGTCCACTTAATAGGGAACT |  |

References

1. Osta WA, Chen Y, Mikhitarian K, Mitas M, Salem M, Hannun YA, et al. EpCAM Is Overexpressed in Breast Cancer and Is a Potential Target for Breast Cancer Gene Therapy. Cancer Res. 2004;64: 5818–5824. doi:10.1158/0008-5472.CAN-04-0754

2. Whyte ML, Smith K, Buchberger A, Luecke LB, Tjan LH, Mori Y, et al. The roseoloviruses downregulate the protein tyrosine phosphatase PTPRC (CD45). Microbiology; 2020 Sep. doi:10.1101/2020.09.29.318709
