# Supplementary material for: Alteration of L-Dopa decarboxylase expression in SARS-CoV-2 infection and its association with the interferon-inducible ACE2 isoform
Source: PLoS One. 2021 Jun 29;16(6):e0253458. doi: 10.1371/journal.pone.0253458 (PMC8241096; doi:10.1371/journal.pone.0253458)
Supplement: S2 Table — (DOCX) [file pone.0253458.s002.docx]

**S2 Table. Demographic data of Influenza positive and negative subjects**

| **Characteristics** | | **Influenza A/B** | |  |
| --- | --- | --- | --- | --- |
|  |  | **Positive** | **Negative** |  |
| **Total Number** | | 38 | 38 |  |
| **Median age in years (IQR)** | | 23 (17-33) | 47(34-68) |  |
| **Age group** | **0-17** | 10 | 4 |  |
|  | **18-39** | 23 | 7 |  |
|  | **40-64** | 4 | 13 |  |
|  | **65+** | 1 | 11 |  |
|  | **NR** | 7 | 3 |  |
| S**ex** | **Male** | 22 | 14 |  |
|  | **Female** | 16 | 23 |  |
|  | **NR** | - | 1 |  |
| **Type of influenza** | **A** | 25 |  |  |
|  | **B** | 13 |  |  |

NR: Data not recorded; IQR: inter-quartile range
