# Supplementary material for: Alteration of L-Dopa decarboxylase expression in SARS-CoV-2 infection and its association with the interferon-inducible ACE2 isoform
Source: PLoS One. 2021 Jun 29;16(6):e0253458. doi: 10.1371/journal.pone.0253458 (PMC8241096; doi:10.1371/journal.pone.0253458)
Supplement: S1 File — (DOCX) [file pone.0253458.s003.docx]

**Supplementary experimental procedures**

**Cell culture**

Human airway epithelial cell line A549 and human hepatic epithelial cell line Huh7.5 [1] (kindly provided by C. Rice, The Rockefeller University) were cultured in Dulbecco’s modified minimal essential medium (DMEM) (Invitrogen). Human monocytic cells THP-1 were grown in RPMI-1640. Mediums were supplemented with 100 U/mL penicillin, 100 µg/mL streptomycin and 10% (v/v) fetal calf serum. To create 3% (v/v) oxygen tension, cells were cultured in a fully humidified incubator supplied with pure nitrogen gas to reduce oxygen as well as with 5% (v/v) CO_2_ at 37 oC (New Brunswick CO_2_ incubator Innova)

**Preparation of virus stocks and infection assays**

DENV virus stocks were generated in VeroE6 cells as described elsewhere [2] and used to inoculate cells for 4 h. HCV virus stocks were generated as described elsewhere [3] and used to infect naive Huh7.5 cells. For infectivity assays, supernatants from the first round of DENV or HCV infection were used to infect naïve cells.

**Whole blood samples collection and analysis**

Whole blood samples were received from Greek patients with positive SARS-CoV-2 RNA PCR and from healthy individuals as control. Samples were collected in tubes containing RNA stabilizing solution (Applied Biosystems, Life Technologies Corporation). Total RNA was isolated from whole blood using the Tempus spin RNA isolation kit (Applied Biosystems, Life Technologies Corporation) and following the manufacturer's instructions. mRNA levels were analyzed by RT-qPCR.

**References**

1. Blight KJ, McKeating JA, Rice CM. Highly permissive cell lines for subgenomic and genomic hepatitis C virus RNA replication. J Virol. 2002;76: 13001–14. doi:10.1128/jvi.76.24.13001-13014.2002

2. Fischl W, Bartenschlager R. High-Throughput Screening Using Dengue Virus Reporter Genomes. In: Gong EY, editor. Antiviral Methods and Protocols. Totowa, NJ: Humana Press; 2013. pp. 205–219. doi:10.1007/978-1-62703-484-5_17

3. Vassilaki N, Friebe P, Meuleman P, Kallis S, Kaul A, Paranhos-Baccala G, et al. Role of the hepatitis C virus core+1 open reading frame and core cis-acting RNA elements in viral RNA translation and replication. J Virol. 2008;82: 11503–15. doi:10.1128/JVI.01640-08
