# Supplementary material for: Alteration of L-Dopa decarboxylase expression in SARS-CoV-2 infection and its association with the interferon-inducible ACE2 isoform
Source: PLoS One. 2021 Jun 29;16(6):e0253458. doi: 10.1371/journal.pone.0253458 (PMC8241096; doi:10.1371/journal.pone.0253458)
Supplement: S2 File — (DOCX) [file pone.0253458.s004.docx]

**S1 Fig.** **XY scatter plot and fitted linear regression lines of DDC, ACE2, dACE2 ISG56 and EPO mRNA expression versus age in SARS-CoV-2-positive (A) and negative (B) groups**. For all genes, no significant correlation with age was observed after calculation of Pearson’s correlation coefficient (-0.1<r< 0.3), p values >0.05.





**S2 Fig.** **Relative ACE2 (A), dACE2 (B), ISG56 (C) and EPO (D) mRNA expression in men as compared to women in SARS-CoV-2 infected** (**right**) **and non-infected groups** (**left**)**.** Data are represented as box plots; line in the middle, median; box edges, 25^th^ to 75^th^ centiles; whiskers, range of values. For all genes, no significant differences were observed between sexes, after calculation of p values with student’s t-test (p>0.05).


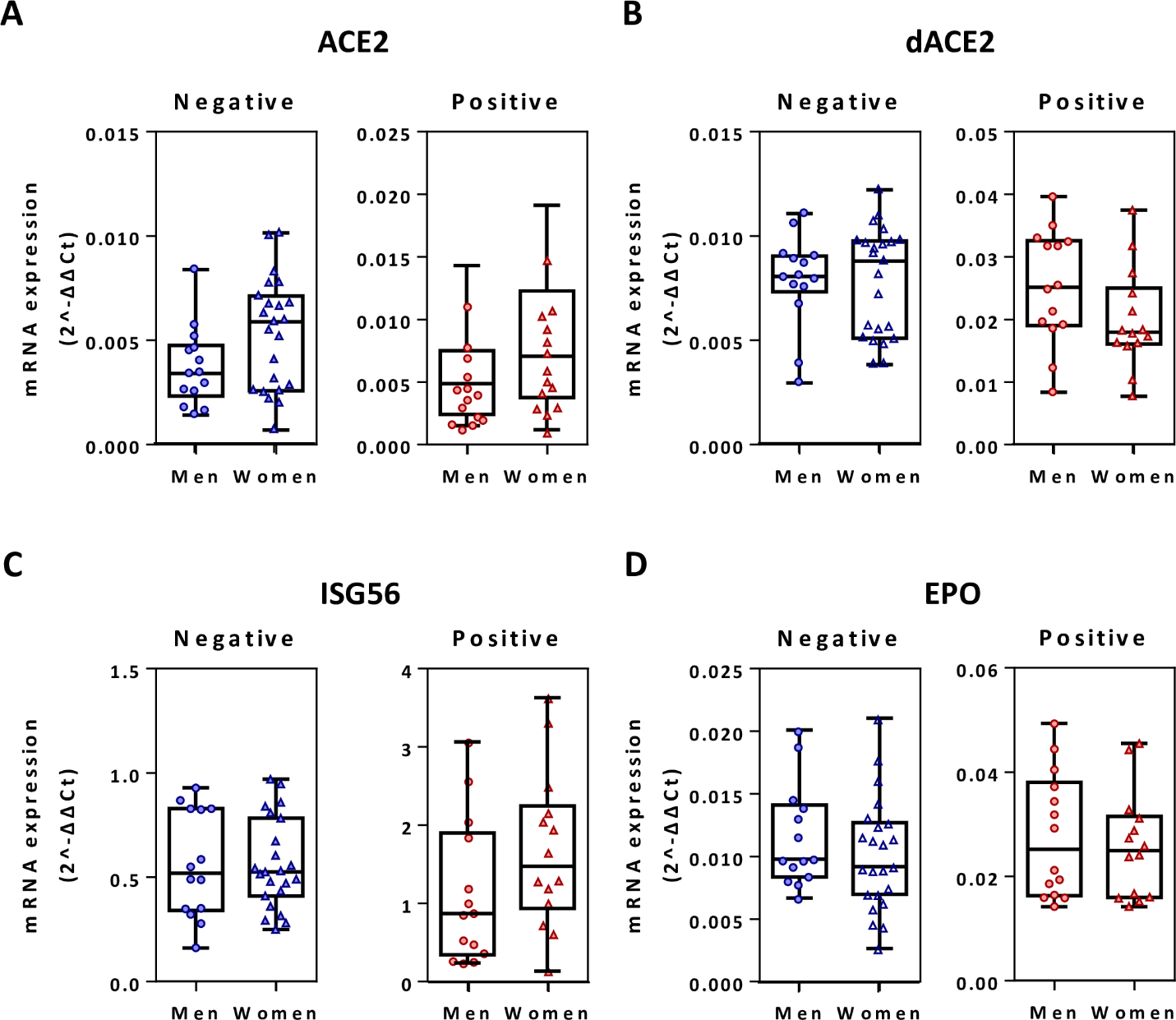


**S3 Fig.** **Relative mRNA expression of epithelial marker EPCAM and immune cell markers CD45, CD74 and LYN in A549 and THP-1 cells.** RT-qPCR analysis of cellular gene mRNAs. YWHAZ mRNA levels were used for normalization. Values obtained from A549 cells were set to one. Mean values from three independent experiments are presented. *p<0.001 (Student’s t test).


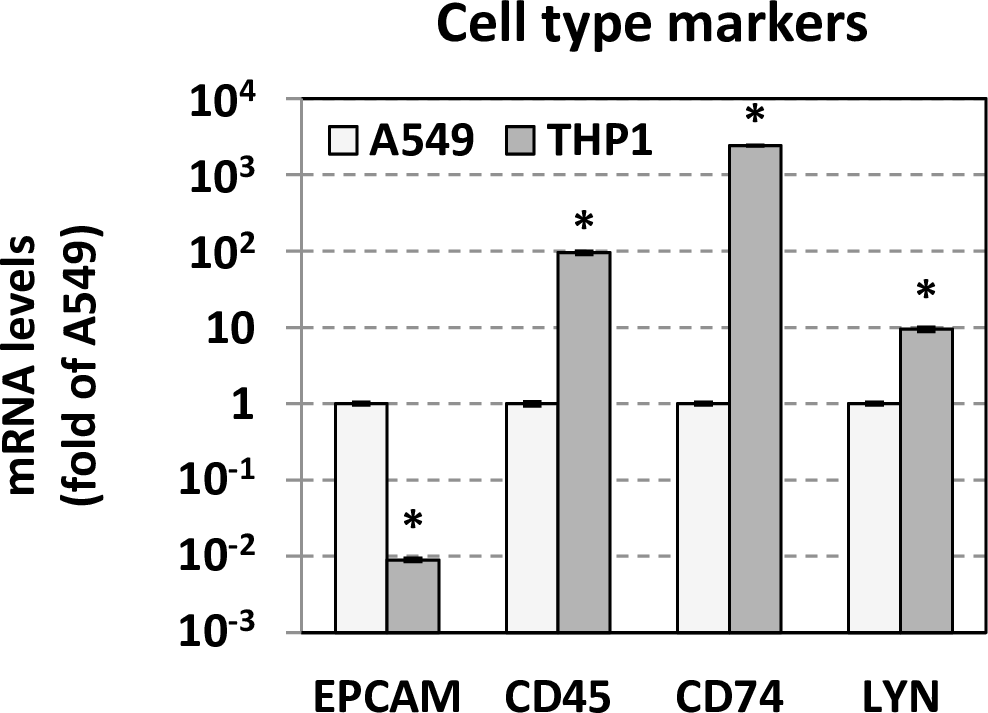


**S4 Fig.** **DDC expression in different cell types of COVID-19 nasopharyngeal swab samples.** Visualization of DDC expression available from Magellan COVID-19 data explorer (<https://digital.bihealth.org>), after analysis of the gene expression data of a single-cell RNA-Seq study performed in 14 COVID-19 patients [1].


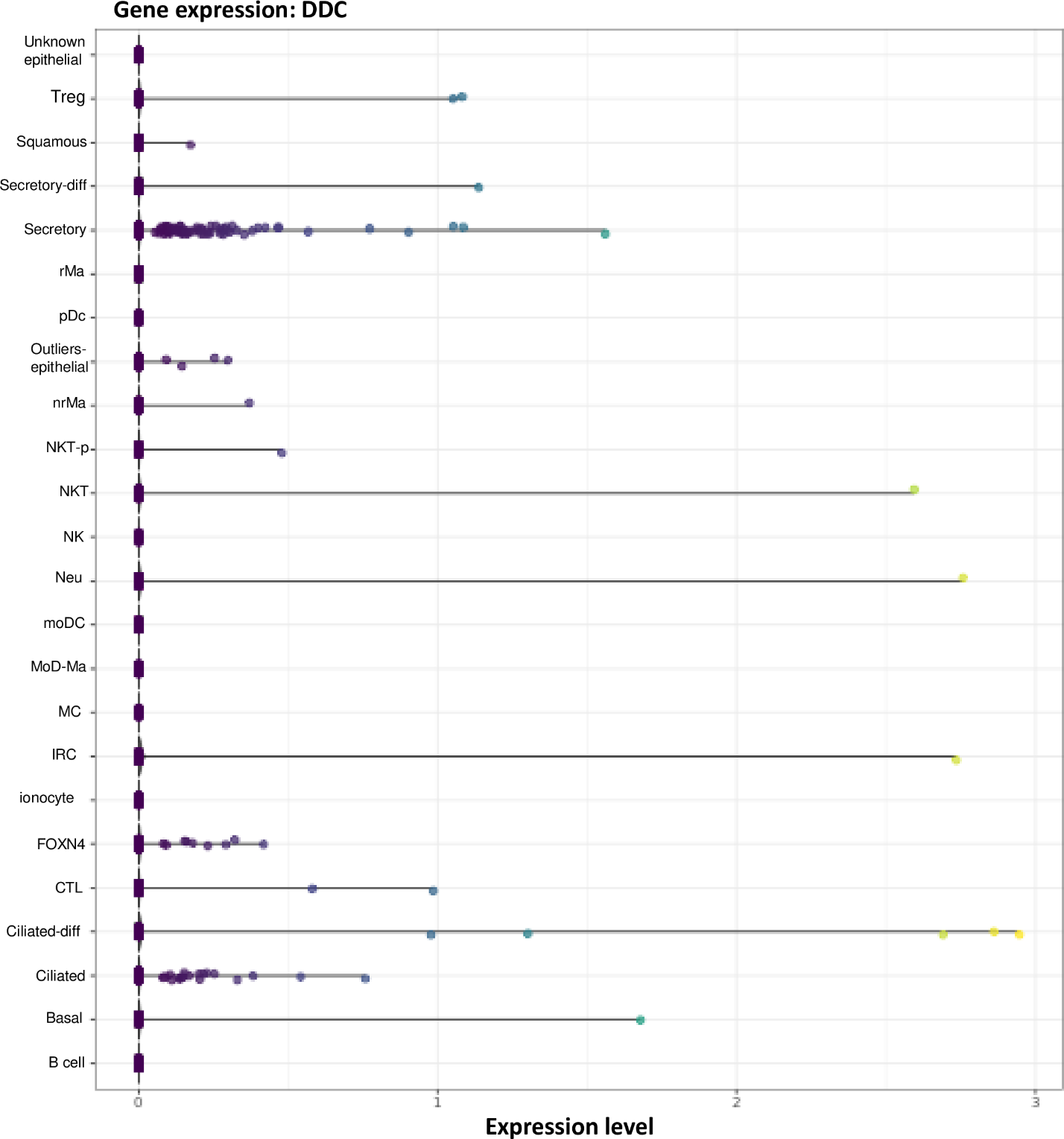


S5 Fig. Evaluation of the correlation of *ISG56* with *ACE2* and *EPO* gene expression levels in SARS-CoV2-positive and negative swab samples. XY scatter plot and fitted linear regression lines of the mRNA levels of (A) ISG56 versus *ACE2* and (B) *EPO* versus *ISG56* in positive (left) and negative (right) samples. Pearson’s or Spearman’s correlation coefficient (*r*) and *p* values (*p*) were calculated.





S6 Fig. Correlation of *DDC*, *dACE2* and *EPO* expression in whole blood samples of SARS-CoV2-infected and non-infected individuals. XY scatter plot and fitted linear regression lines of the mRNA levels of (A) *DDC* versus *dACE2*, (B) *DDC* versus *EPO*, and (C) *EPO* versus *dACE2*, in positive (left) and negative (right) samples. Pearson’s correlation coefficient (*r*) and *p* values (*p*) were calculated.





**S7 Fig. Downregulation of *DDC* and *EPO* 24 hours post-infection with SARS-CoV-2 of A549, A549 overexpressing ACE2 and bronchial epithelial cells Calu-3**. Further analysis of RNA-seq gene expression data of Blanco-Melo et al. [2] that are available in <http://rstats.immgen.org/Skyline_COVID-19/skyline.html>. *GLUT1* and *VEGFA* expression was also analyzed to monitor virus-induced hypoxia signaling. MI: mock-infected, I:infected cells.


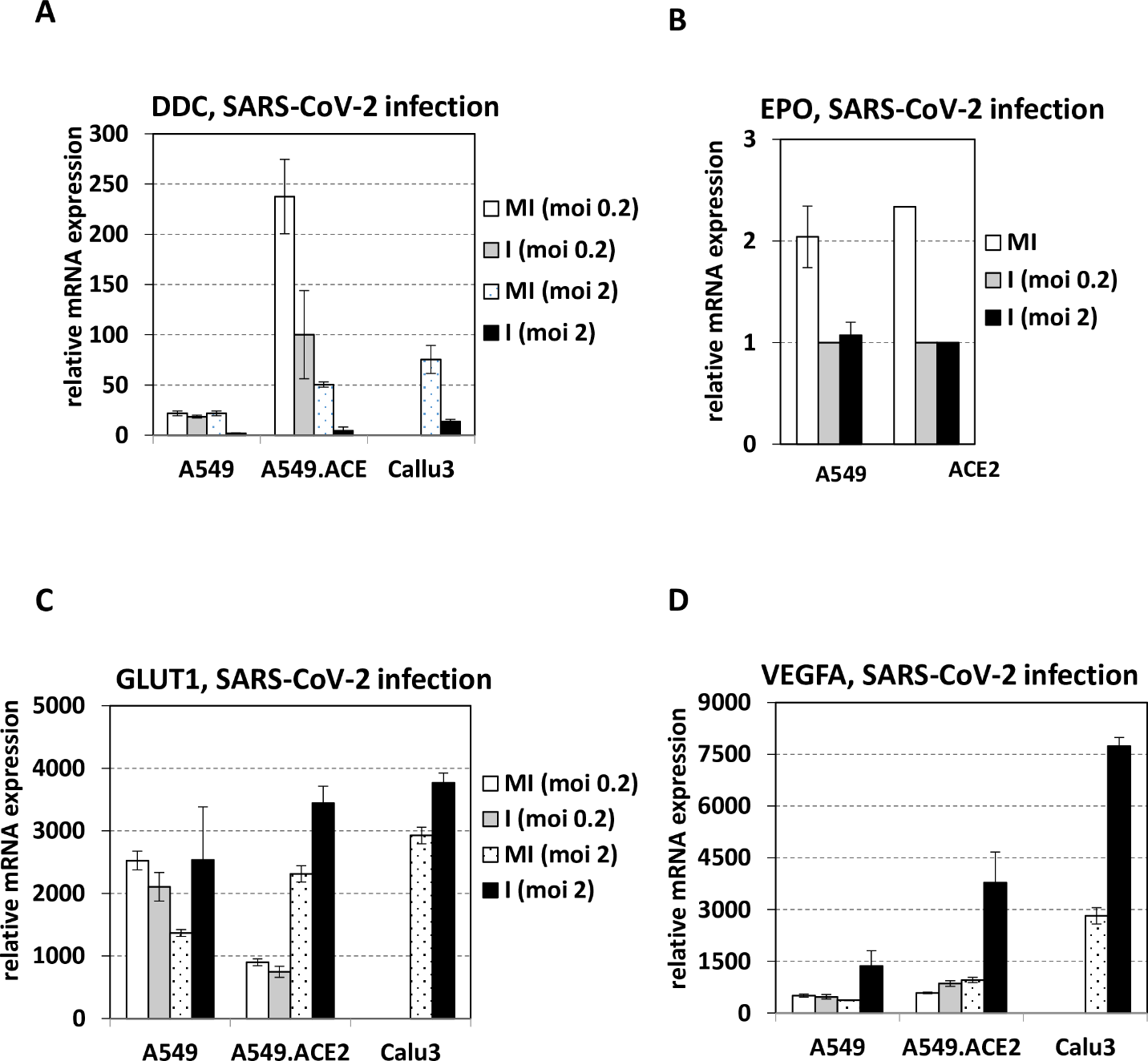


**S8 Fig.** **Cytopathic effect (CPE) of SARS-CoV2 in VeroE6** 48 hours post-infection (h.p.i) monitored using inverted phase contract microscopy. Mock-infected cells were used as negative control.


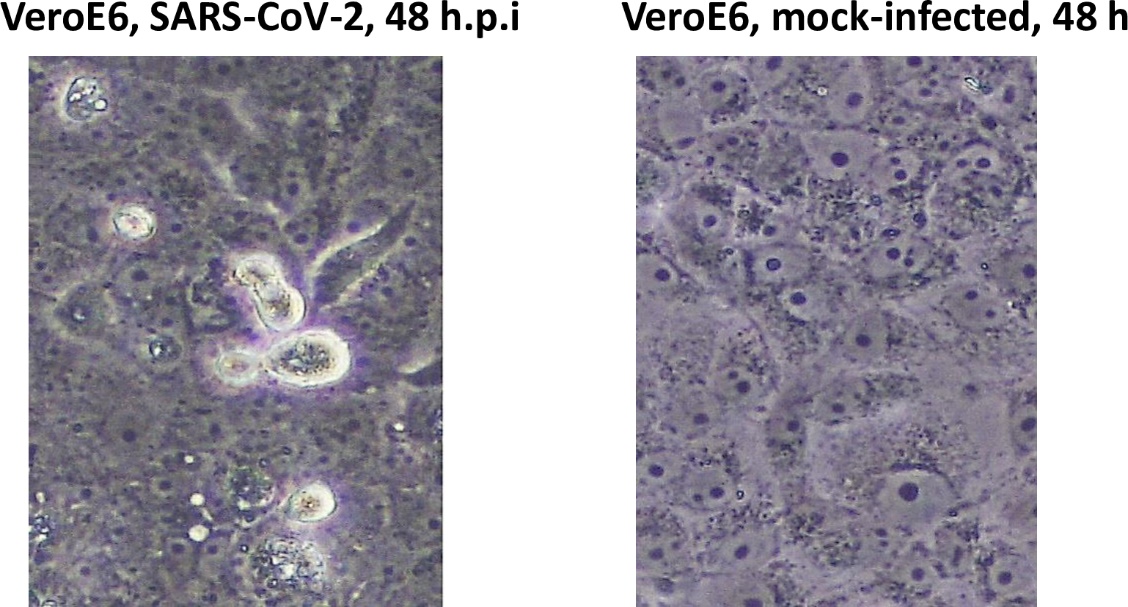


**S9 Fig. (A)** **Effect of hypoxia on *DDC*, *dACE2*, *ACE2*, *ISG56*, *EPO* and GLUT1 expression in Huh7.5 hepatic epithelial cells.** Huh7.5 cells [3] were cultured under atmospheric (20% v/v) or hypoxic (3% v/v) oxygen tension for 48 h. Cell lysates were analyzed using RT-qPCR. YWHAZ mRNA levels were used for normalization. Bars represent mean values from three independent experiments in triplicates. Error bars indicate standard deviations. *p<0.001 vs 20%-cultured cells (Student’s t test) **(B, C) Effect of Dengue (DENV) and HCV viruses in *DDC*, *dACE2*, *ACE2, EPO* and *ISG56* gene expression in cell culture-infected cells.** Huh7.5 cells were infected with (B) DENV2 16681 strain [4] or (C) Jc1 virus [5] carrying the full-length HCV genome (kindly provided by Prof. R. Bartenschlager, Heidelberg University). Viral infections were performed at an m.o.i of 1 for 48 h (DENV) or 96 h (Jc1). Mock-infected (MI) cells were cultured in parallel, as control. Cell lysates were analyzed using RT-qPCR. YWHAZ mRNA levels were used for normalization. Bars represent mean values from three independent experiments in triplicates. Error bars indicate standard deviations. *p<0.001 vs MI cells (Student’s t test).


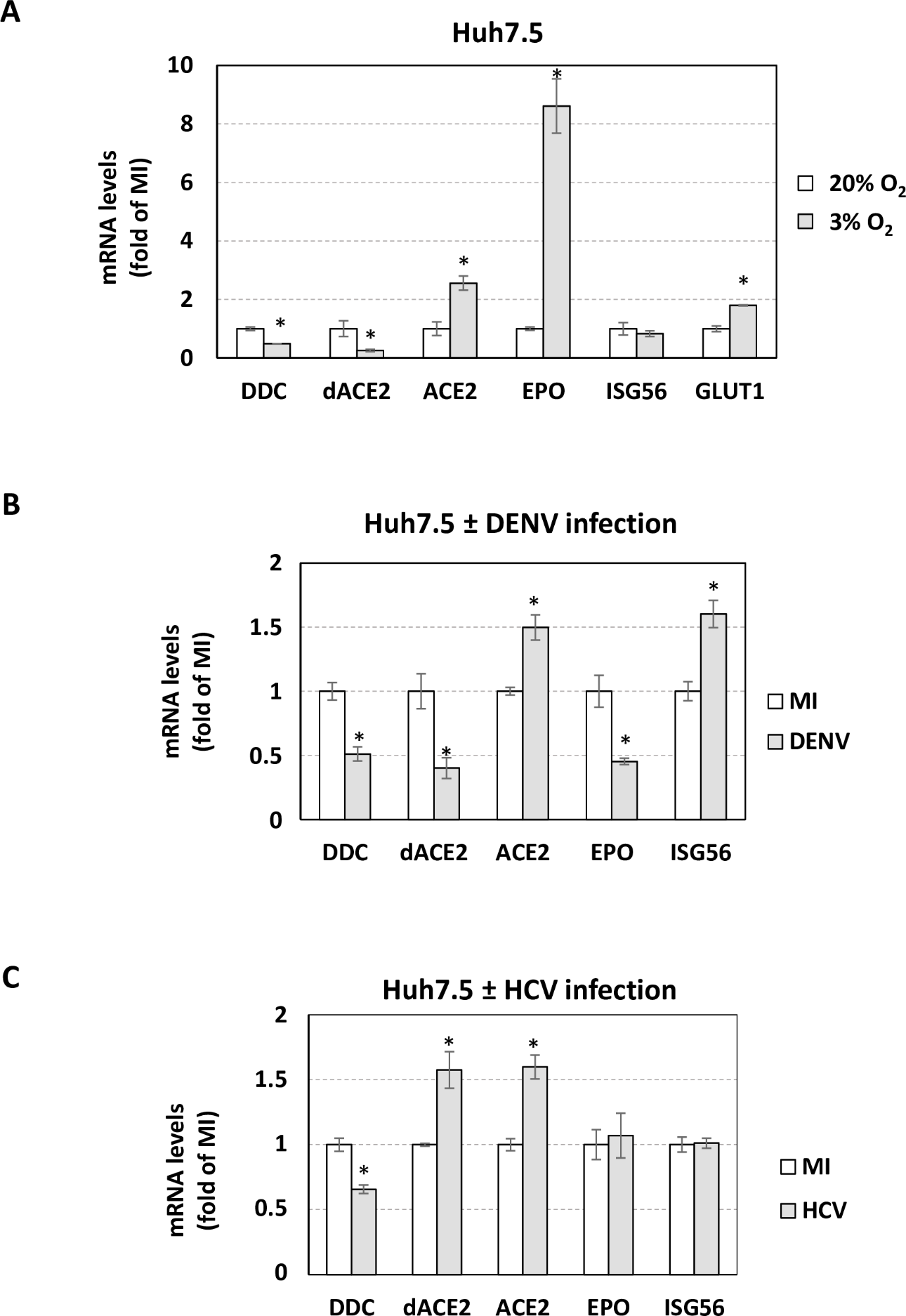


**Supplementary References**

1. Chua RL, Lukassen S, Trump S, Hennig BP, Wendisch D, Pott F, et al. COVID-19 severity correlates with airway epithelium-immune cell interactions identified by single-cell analysis. Nat Biotechnol. 2020;38: 970–979. doi:10.1038/s41587-020-0602-4

2. Blanco-Melo D, Nilsson-Payant BE, Liu WC, Uhl S, Hoagland D, Moller R, et al. Imbalanced Host Response to SARS-CoV-2 Drives Development of COVID-19. Cell. 2020;181: 1036-1045 e9. doi:10.1016/j.cell.2020.04.026

3. Blight KJ, McKeating JA, Rice CM. Highly permissive cell lines for subgenomic and genomic hepatitis C virus RNA replication. J Virol. 2002;76: 13001–14. doi:10.1128/jvi.76.24.13001-13014.2002

4. Fischl W, Bartenschlager R. High-Throughput Screening Using Dengue Virus Reporter Genomes. In: Gong EY, editor. Antiviral Methods and Protocols. Totowa, NJ: Humana Press; 2013. pp. 205–219. doi:10.1007/978-1-62703-484-5_17

5. Reiss S, Rebhan I, Backes P, Romero-Brey I, Erfle H, Matula P, et al. Recruitment and activation of a lipid kinase by hepatitis C virus NS5A is essential for integrity of the membranous replication compartment. Cell Host Microbe. 2011;9: 32–45. doi:10.1016/j.chom.2010.12.002
